# Diagnostic Utility of Non-invasive Tests for Inflammatory Bowel Disease: An Umbrella Review

**DOI:** 10.3389/fmed.2022.920732

**Published:** 2022-07-11

**Authors:** Jin-Tong Shi, Yuexin Zhang, Yuehan She, Hemant Goyal, Zhi-Qi Wu, Hua-Guo Xu

**Affiliations:** ^1^Department of Laboratory Medicine, The First Affiliated Hospital of Nanjing Medical University, Nanjing, China; ^2^Branch of National Clinical Research Center for Laboratory Medicine, Nanjing, China; ^3^Department of Medicine, The Wright Center for Graduate Medical Education, Scranton, PA, United States

**Keywords:** inflammatory bowel disease, non-invasive tests, diagnostic performance, Crohn’s disease, ulcerative colitis

## Abstract

**Background:**

This study aims to consolidate evidence from published systematic reviews and meta-analyses evaluating the diagnostic performances of non-invasive tests for inflammatory bowel disease (IBD) in various clinical conditions and age groups.

**Methods:**

Two independent reviewers systematically identified and appraised systematic reviews and meta-analyses assessing the diagnostic utility of non-invasive tests for IBD. Each association was categorized as adults, children, and mixed population, based on the age ranges of patients included in the primary studies. We classified clinical scenarios into diagnosis, activity assessment, and predicting recurrence.

**Results:**

In total, 106 assessments from 43 reviews were included, with 17 non-invasive tests. Fecal calprotectin (FC) and fecal lactoferrin (FL) were the most sensitive for distinguishing IBD from non-IBD. However, anti-neutrophil cytoplasmic antibodies (ANCA) and FL were the most specific for it. FC and FL were the most sensitive and specific tests, respectively, to distinguish IBD from irritable bowel syndrome (IBS). Anti-*Saccharomyces cerevisiae* antibodies (ASCA), IgA, were the best test to distinguish Crohn’s disease (CD) from ulcerative colitis (UC). Interferon-γ release assay was the best test to distinguish CD from intestinal tuberculosis (ITB). Ultrasound (US) and magnetic resonance enterography (MRE) were both sensitive and specific for disease activity, along with the high sensitivity of FC. Small intestine contrast ultrasonography (SICUS) had the highest sensitivity, and FC had the highest specificity for operative CD recurrence.

**Conclusion:**

In this umbrella review, we summarized the diagnostic performance of non-invasive tests for IBD in various clinical conditions and age groups. Clinicians can use the suggested non-invasive test depending on the appropriate clinical situation in IBD patients.

## Introduction

Inflammatory bowel diseases (IBD) [Crohn’s disease (CD) and ulcerative colitis (UC)] are idiopathic disorders causing inflammation of the gastrointestinal tract. IBD is emerging as a globally important disease with increasing incidence. Although incidence has started to relatively stabilize in western countries, the disease burden remains high as prevalence surpasses 0.3% (1).

Gastrointestinal endoscopy has remained a reference standard but invasive test for the diagnosis, management, prognostics, and surveillance of IBD. However, endoscopy can be associated with considerable cost, risk, and burden to patients and healthcare systems, and it is the lowest acceptable tool for patients (2).

Accurate non-invasive tests such as biomarkers and radiological examinations would be ideal (3, 4). Several promising non-invasive tests that could fulfill this role, including fecal calprotectin (FC) (5) and ultrasound (US) (6), have been studied. Despite many studies assessing the diagnostic performance of non-invasive tests for IBD, to the best of our knowledge, there has been no systematic effort to summarize and critically appraise this body of evidence. Therefore, we performed an umbrella review of meta-analyses, based on different clinical conditions (including diagnosis, activity assessment, and recurrence) and age groups (children, adults, and mixed population), to provide a comprehensive synopsis of the diagnostic performance and validity of reported non-invasive tests for IBD.

## Methods

### Search Strategy

Two reviewers (J-TS and Z-QW) independently searched PubMed, Embase, Web of Science, and Cochrane Library databases from inception to 16 April 2020. The search was limited to systematic reviews and meta-analyses without language restrictions. Supplementary Appendix 1 provides a detailed search strategy.

### Study Selection and Data Extraction

Systematic reviews or meta-analyses meeting the following criteria were included: it described the conduct of the systematic review in adequate detail, and an attempt was made to identify all of the relevant primary studies in at least one database with provided search strategy and quality appraisal of the primary studies (7). Guidelines, narrative reviews, literature reviews, genetic studies, protocol, conference abstracts, and reviews assessing scoring indices were excluded.

Two reviewers (J-TS and Z-QW) independently carried out the study selection and data extraction from the eligible articles. Extracted data included author, year of publication, number of participants, number and type of studies included, appraisal instrument used, reference standard, outcomes assessed, heterogeneity, and study findings.

### Quality Assessment

The methodological quality of included reviews was assessed independently by J-TS and Z-QW using the online AMSTAR 2 (A Measurement Tool to Assess Systematic Reviews) checklist (8). AMSTAR 2 is a validated and reliable quality measurement tool for systematic reviews, with 16 domains. Seven of these domains are considered critical. Shortcomings in any of the critical domains could affect the overall validity of a review. It results in an assessment of the methodologic quality as 1 of 2 grades: high, moderate, low, or critically low (9).

### Identification of Age Groups

Based on the age ranges of primary studies included, associations can be categorized as adults, children, and mixed population. We defined children as under the age of 18 years (10). If a systematic review purporting to assess the accuracy in adults included people younger than 18 years, it would be classified as a mixed population. Supplementary Appendix 2 presents the process of identifying age groups.

### Overlapping and Outdated Associations

Associations in two or more reviews overlapped if they evaluated the same test in the same clinical condition and same age group. Incorporating results of overlapping reviews could lead to double inclusion resulting in biased findings and estimates (11, 12). In addition, up to 50% of published systematic reviews were considered out of date after 5.5 years. Therefore, we categorized overlapping systematic reviews as outdated (published before October 2015) and contemporary (published after October 2015).

For contemporary reviews found to have overlapping assessments, we generated a graphical cross-tabulation (citation matrix) of the overlapping reviews (in columns) and the included primary studies (in rows) (13). Corrected covered area (CCA) was a validated method to quantify the degree of overlap between two or more reviews. We used a citation matrix to calculate CCA. According to CCA, the overlap can be categorized as very high (CCA > 15%), high (CCA 11–15%), moderate (CCA 6–10%), or slight (CCA 0–5%) (14).

In all the systematic reviews that met the inclusion criteria, all non-overlapping reviews were included. A rigorous management tool was used for the overlapping reviews. Supplementary Appendix 3 shows the citation matrices for all overlapping studies. Supplementary Appendix 4 presents the management of overlapping reviews.

### Data Synthesis

Systematic reviews that met the inclusion criteria formed the unit of analysis. Only data available from systematic reviews were presented. Results from systematic reviews were synthesized with a narrative synthesis, with a tabular presentation of findings and forest plots for studies that performed a meta-analysis. Summary tables describing review characteristics and findings were also presented.

### Update of Eligible Reviews

We used the framework recommended by Garner et al. (15) to determine whether an update was necessary. An existing review qualified for an update if all of the following were met:

• The review achieved at least a moderate rating with the AMSTAR 2 quality assessment tool (9).
• A focused or abbreviated search of primary studies using the key search terms from the search strategy of an existing review to identify newly published studies that met the inclusion criteria of the review.
• The findings from newly published studies would change the conclusion or credibility of the review.

Supplementary Appendix 5 describes the search strategy used to identify newly published studies. YXZ and YHS initially screened the eligible newly published studies. Full-text screening and data extracting were accomplished by JTS and ZQW.

With findings from newly published studies, we relied on statistical methods using the bivariate model (16) to pool the data from newly published studies with the data from the original meta-analysis (17) (for meta-analyses) and discussion with senior authors (for reviews without meta-analyses) to determine whether a full update of the existing review was needed (18).

If an update was considered necessary, the original methods used in the conduct of the existing review were replicated. Supplementary Appendix 6 summarizes the evaluation process for considering reviews for updates adapted from Ahmadzai et al. (19).

## Results

### Literature Search

The search retrieved 1,897 articles. After removing duplicates and screening titles and abstracts, 113 articles qualified for full-text screening. Seven outdated reviews were further excluded. Finally, 46 reviews were included. Supplementary Appendix 7 summarizes the study selection process with accurate numbers of studies. Supplementary Appendix 8 provides the list of excluded studies with reasons for exclusion.

### Methodological Quality

Twenty-two reviews (5, 6, 10, 20–38) were rated as moderate in quality, and twenty-three reviews (39–60) were rated as low, while one review (61) was rated as critically low in quality (Supplementary Appendix 9). In the seven critical domains, most low-quality reviews had not stated that the methods were established before conducting the study.

### Overlapping and Non-overlapping Assessment

Seventeen reviews reported overlapping assessment (5, 6, 29, 32, 36, 37, 46, 49–52, 54, 58, 59, 61–63). Supplementary Appendix 10 describes the general characteristics of overlapping reviews, including the decision to retain or exclude an assessment. Supplementary Appendix 3 provides the citation matrices for overlapping reviews to assess the degree of overlap. Supplementary Appendix 11 lists forty-six reviews with non-overlapping assessments that were included and one contemporary review that was excluded because of overlap.

### Study Characteristics of Reviews With Non-overlapping Assessments

Non-invasive tests for IBD assessed in the included reviews were FC, C-reactive protein (CRP), erythrocyte sedimentation rate (ESR), platelet count (PLT), hemoglobin (Hb), albumin (Alb), ASCA, anti-neutrophil cytoplasmic antibodies (ANCA), fecal lactoferrin (FL), US, computed tomography (CT), magnetic resonance imaging enterography (MRE), scintigraphy, autoantibodies-to-glycoprotein-2 (AntiGP2), interferon-γ release assays (IGRA), fecal immunochemical (FIT), microRNA, and S100A12. Of the 46 reviews included, 43 conducted meta-analyses. Supplementary Table 1 summarizes the general characteristics of the reviews and meta-analyses included in the umbrella review.

### Summary Findings

**FIGURE 1:**
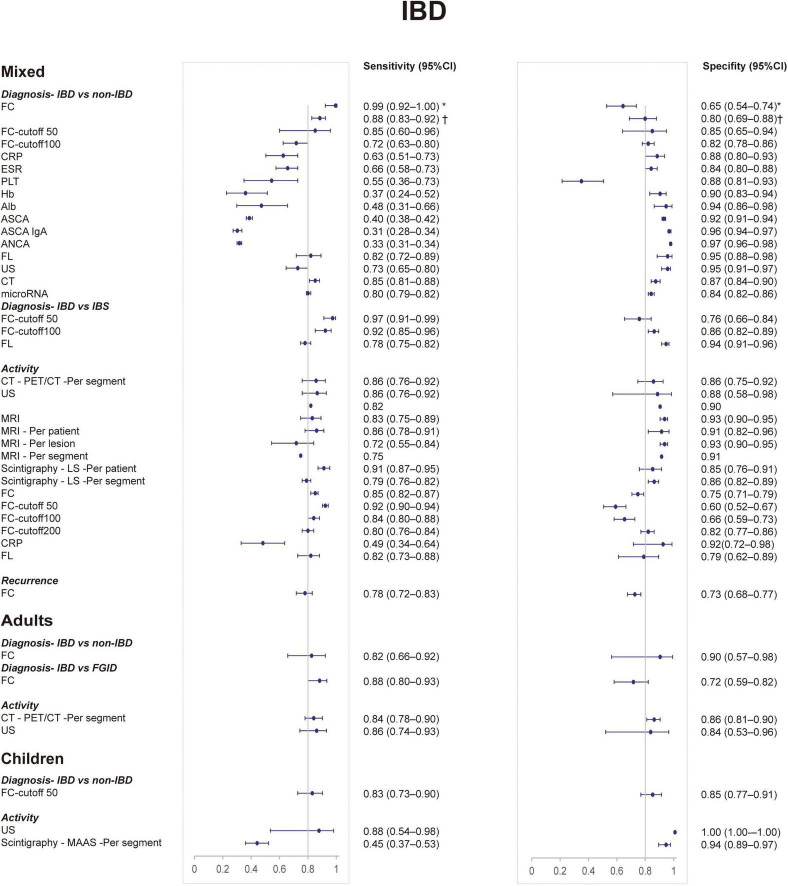
Forest plots of sensitivity and specificity of non-invasive tests for inflammatory bowel disease from meta-analyses. CI, confidence interval. *Age range: 0.8–19.9, ^†^age range: 14–90.

**FIGURE 2:**
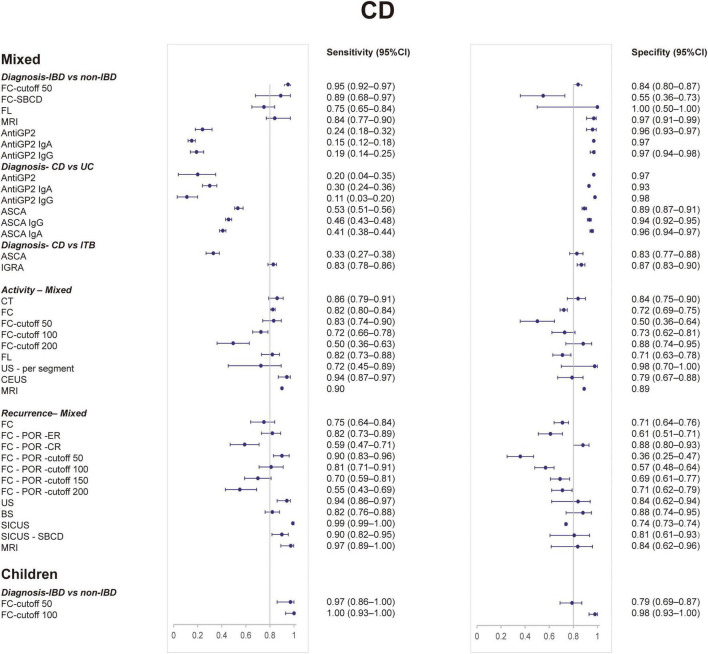
Forest plots of sensitivity and specificity of non-invasive tests for Crohn’s disease from meta-analyses. CI, confidence interval.

**FIGURE 3:**
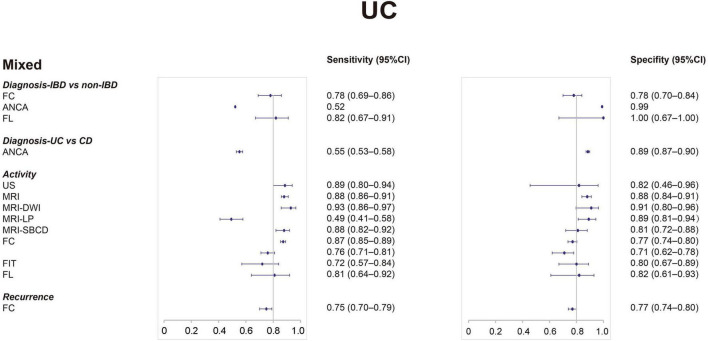
Forest plots of sensitivity and specificity of non-invasive tests for ulcerative colitis from meta-analyses. CI, confidence interval.

Table 1 shows the diagnostic utility of non-invasive tests for IBD in different clinical scenarios and age groups. Tables 2, 3 show the diagnostic utility of non-invasive tests for CD and UC, respectively. The clinical scenarios include diagnosis (IBD vs. non-IBD), diagnosis (IBD vs. IBS), diagnosis (IBD vs. FGID, functional gastrointestinal disorders), diagnosis (CD vs. ITB, intestinal tuberculosis), diagnosis (CD vs. UC), activity assessment, and recurrence. Figure 1 presents the forest plots of sensitivity (Se) and specificity (Sp) of non-invasive tests for IBD. Figures 2, 3 present the forest plots for CD and UC, respectively. Supplementary Tables 2, 3 show the findings of meta-analyses and narrative synthesis from systematic reviews.

**TABLE 1:** Summary findings for each non-invasive tests and diagnostic performance (IBD).

Non-invasive tests		Diagnostic performance (95% CI)		
		Sensitivity	Specificity	AUC
**Mixed**				
***Diagnosis- IBD vs. non-IBD***				
FC	FC	0.99 (0.92–1.00) * 0.882 (0.827–0.921) †	0.65 (0.54–0.74) * 0.799 (0.693–0.875) †	NA
	Cut-off 50μg/g	0.850 (0.605–0.955)	0.847 (0.647–0.943)	NA
	Cut-off 100μg/g	0.72 (0.63–0.80)	0.82 (0.78–0.86)	NA
CRP		0.63 (0.51–0.73)	0.88 (0.80–0.93)	NA
ESR		0.66 (0.58–0.73)	0.84 (0.80–0.88)	NA
PLT		0.55 (0.36–0.73)	0.88 (0.81–0.93)	NA
Hb		0.37 (0.24–0.52)	0.90 (0.83–0.94)	NA
Alb		0.48 (0.31–0.66)	0.94 (0.86–0.98)	NA
ASCA	ASCA	0.397 (0.376–0.418)	0.925 (0.913–0.937)	0.783
	IgA	0.314 (0.285–0.345)	0.96 (0.943–0.973)	0.821
ANCA		0.328 (0.312–0.344)	0.971 (0.964–0.977)	0.872
FL		0.82 (0.72–0.89)	0.95 (0.88–0.98)	0.95 (0.93–0.97)
US		0.73 (0.65–0.80)	0.95 (0.91–0.97)	NA
CT- per segment		0.85 (0.81–0.88)	0.87 (0.84–0.90)	0.933
microRNA		0.80 (0.79–0.82)	0.84 (0.82–0.86)	0.89
***Diagnosis- IBD vs. IBS***				
FC	Cut-off 50μg/g	0.97 (0.91–0.99)	0.76 (0.66–0.84)	NA
	Cut-off 100μg/g	0.92 (0.85–0.96)	0.86 (0.82–0.89)	NA
FL		0.78 (0.75–0.82)	0.94 (0.91–0.96)	0.94
***Activity***				
CT-Per segment		0.856 (0.76–0.92)	0.855 (0.75–0.92)	NA
US-Per segment		0.864 (0.761–0.927) 0.82	0.883 (0.581–0.976) 0.9	NA 0.90 (0.75–1.00)
MRE	MRE	0.83 (0.75–0.89)	0.93 (0.90–0.95)	0.95 (0.93–0.97)
	Per-patient	0.86 (0.78–0.91)	0.91 (0.82–0.96)	NA
	Per-lesion	0.72 (0.55–0.84)	0.93 (0.90–0.95)	NA
	Per-segment	0.75	0.91	0.88 (0.82–0.93)
Scintigraphy	LS-per patient	0.91 (0.87–0.95)	0.85 (0.76–0.91)	NA
	LS-per segment	0.79 (0.76–0.82)	0.86 (0.82–0.89)	NA
FC	FC	0.85 (0.82–0.87)	0.75 (0.71–0.79)	NA
	Cut-off 50μg/g	0.92 (0.90–0.94)	0.60 (0.52–0.67)	NA
	Cut-off 100μg/g	0.84 (0.80–0.88)	0.66 (0.59–0.73)	NA
	Cut-off 250μg/g	0.80 (0.76–0.84)	0.82 (0.77–0.86)	NA
CRP		0.49 (0.34–0.64)	0.92 (0.72–0.98)	0.72 (0.68–0.76)
FL		0.82 (0.73–0.88)	0.79 (0.62–0.89)	0.87 (0.84–0.90)
***Recurrence***				
FC		0.78 (0.72–0.83)	0.73 (0.68–0.77)	0.83

**Adults**				
***Diagnosis- IBD vs. non-IBD***				
FC		0.825 (0.661–0.920)	0.900 (0.573–0.984)	NA
***Diagnosis- IBD vs. FGID***				
FC		0.88 (0.80–0.93)	0.72 (0.59–0.82)	0.89
***Activity***				
CT-Per segment		0.84 (0.78–0.90)	0.86 (0.81–0.90)	NA
US-Per segment		0.860 (0.745–0.928)	0.836 (0.533–0.958)	NA

**Children**				
***Diagnosis-IBD vs. non-IBD***				
FC	FC	NA	NA	0.95 (0.93–0.98)
	Cut-off 50μg/g	0.83 (0.73–0.90)	0.85 (0.77–0.91)	0.96
CRP		NA	NA	0.79 (0.73–0.85)
ESR		NA	NA	0.84 (0.82–0.87)
PLT		NA	NA	0.79 (0.75–0.83)
Hb		NA	NA	0.76 (0.71–0.80)
Alb		NA	NA	0.82 (0.73–0.90)
***Activity***				
US		0.876 (0.542–0.977)	1.0	NA
Scintigraphy-MAAS-per segment		0.45 (0.37–0.53)	0.94 (0.89–0.97)	NA

NA, not available, *age range: 0.8–19.9, †: age range: 14–90. FC, fecal calprotectin; CRP, C-reactive protein; ESR, erythrocyte sedimentation rate; PLT, platelet count; Hb, hemoglobin; Alb, albumin; ASCA, Anti-Saccharomyces cerevisiae antibodies; ANCA, anti-neutrophil cytoplasmic antibodies; FL, fecal lactoferrin; US, Ultrasound; CT, computed tomography; MRE, magnetic resonance imaging enterography.

**TABLE 2:** Summary findings for each non-invasive tests and diagnostic performance (CD).

Non-invasive tests		Diagnostic performance (95% CI)		
		Sensitivity	Specificity	AUC
**Mixed**				
***Diagnosis- IBD vs. non-IBD***				
FC	Cut-off 50μg/g	0.95 (0.92–0.97)	0.84 (0.80–0.87)	NA
	SBCD	0.89 (0.68–0.97)	0.55 (0.36–0.73)	NA
FL		0.75 (0.65–0.84)	1.00 (0.50–1.00)	0.84 (0.81–0.87)
MRI-SBCD		0.84 (0.77–0.90)	0.97 (0.91–0.99)	0.95
AntiGP2	AntiGP2	0.24 (0.18–0.32)	0.96 (0.93–0.97)	0.72 (0.68–0.76)
	IgA	0.15 (0.12–0.18)	0.97 (median)	NA
	IgG	0.19 (0.14–0.25)	0.97 (0.94–0.98)	0.71 (0.67–0.75)
***Diagnosis- CD vs. UC***				
AntiGP2	AntiGP2	0.20 (0.04–0.35)	0.97 (median)	NA
	IgA	0.11 (0.03–0.20)	0.98 (median)	NA
	IgG	0.30 (0.24–0.36)	0.93 (median)	NA
ASCA	ASCA	0.533 (0.508–0.557)	0.892 (0.872–0.910)	0.836
	IgA	0.408 (0.381–0.435)	0.955 (0.938–0.967)	0.863
	IgG	0.457 (0.432–0.483)	0.935 (0.917–0.949)	0.85
***Diagnosis- CD vs. ITB***				
ASCA		0.33 (0.27–0.38)	0.83 (0.77–0.88)	0.58
IGRA		0.828 (0.784–0.855)	0.867 (0.832–0.896)	NA
***Activity***				
CT-SBCD-per patient		0.86 (0.79–0.91)	0.84 (0.75–0.90)	NA
FC	FC	0.824 (0.802–0.844)	0.721 (0.69–0.75)	0.84
	Cut-off 50μg/g	0.831 (0.740–0.895)	0.502 (0.359–0.644)	0.774
	Cut-off 100μg/g	0.725 (0.657–0.784)	0.728 (0.622–0.814)	0.763
	Cut-off 200μg/g	0.495 (0.361–0.629)	0.882 (0.738–0.952)	0.67
FL		0.82 (0.73–0.88)	0.71 (0.63–0.78)	0.84 (0.80–0.87)
MRI		0.9	0.89	NA
US	Per segment	0.725 (0.454–0.894)	0.977 (0.700–0.999)	NA
	CEUS	0.94 (0.87–0.97)	0.79 (0.67–0.88)	0.94
***Recurrence***				
FC	FC	0.75 (0.64–0.84)	0.71 (0.64–0.76)	0.79
	POR-ER	0.82 (0.73–0.89)	0.61 (0.51–0.71)	0.77 (0.74–0.81)
	POR-CR	0.59 (0.47–0.71)	0.88 (0.80–0.93)	0.97
	POR-Cut-off 50μg/g	0.90 (0.83–0.96)	0.36 (0.25–0.47)	0.72
	POR-Cut-off 100μg/g	0.81 (0.71–0.91)	0.57 (0.48–0.64)	0.67
	POR-Cut-off 150μg/g	0.70 (0.59–0.81)	0.69 (0.61–0.77)	0.73
	POR-Cut-off 200μg/g	0.55 (0.43–0.69)	0.71 (0.62–0.79)	0.69
US	POR	0.94 (0.86–0.97)	0.84 (0.62–0.94)	0.9
	POR-BS	0.82 (0.76–0.88)	0.88 (0.74–0.95)	0.875
	POR-SICUS	0.99 (0.99–1.00)	0.74 (0.73–0.74)	0.92
	POR-SBCD–SICUS	0.899 (0.817–0.953)	0.808 (0.606–0.934)	NA
MRI-POR		0.973 (0.891–0.998)	0.837 (0.616–0.959)	0.9767

**Children**				
***Diagnosis- IBD vs. non-IBD***				
FC	Cut-off 50μg/g	0.97 (0.86–1.00)	0.79 (0.69–0.87)	NA
	Cut-off 100μg/g	1.00 (0.93–1.00)	0.98 (0.93–1.00)	NA

**TABLE 3:** Summary findings for each non-invasive tests and diagnostic performance (UC).

Non-invasive tests		Diagnostic performance (95% CI)*		
		Sensitivity	Specificity	AUC
**Mixed**				
***Diagnosis- IBD vs. non-IBD***				
FC		0.78 (0.69–0.86)	0.78 (0.70–0.84)	NA
ANCA		0.522	0.99	NA
FL		0.82 (0.67–0.91)	1.00 (0.67–1.00)	0.94 (0.91–0.96)
***Diagnosis- UC vs. CD***				
ANCA		0.553 (0.530–0.576)	0.885 (0.871–0.898)	0.818
***Activity***				
US-per segment		0.886 (0.800–0.939)	0.819 (0.456–0.961)	NA
MRE	MRE	0.88 (0.86–0.91)	0.88 (0.84–0.91)	0.93
	DWI-per segment	0.929 (0.858–0.966)	0.910 (0.797–0.963)	NA
	LP	0.493 (0.410–0.578)	0.891 (0.813–0.944)	0.82
	SBCD-per patient	0.88 (0.82–0.92)	0.81 (0.72–0.88)	0.91
FC		0.873 (0.854–0.891) * 0.76 (0.71–0.81) †	0.771 (0.737–0.803) * 0.71 (0.62–0.78) †	0.91* 0.79 (0.75–0.82) †
FIT		0.72 (0.57–0.84)	0.80 (0.67–0.89)	NA
FL		0.81 (0.64–0.92)	0.82 (0.61–0.93)	0.88 (0.85–0.91)
***Recurrence***				
FC		0.75 (0.70–0.79)	0.77 (0.74–0.80)	0.82

NA, not available; *: endoscopic activity as reference; †: histological activity as reference.

### Diagnosis: Inflammatory Bowel Disease vs. Non-inflammatory Bowel Disease

#### Mixed Population

For IBD, FC was the most sensitive test with a sensitivity of 0.99 (0.92–1.00) (46). ANCA showed the highest specificity 0.971 (0.964–0.977) (20). The sensitivity and specificity of CT, FL, and microRNA were both balanced (41, 44). The other tests performed well in specificity but poorly in sensitivity, including US, ESR, CRP, PLT, Alb, Hb, and ASCA (20, 46).

For UC, FL had both the best sensitivity (0.82; 0.67–0.91) and the best specificity (1.00; 0.67–1.00) (44). The other biomarkers were FC (Se, 0.78; 0.69–0.86/Sp, 0.78; 0.70–0.84) (21), and ANCA (Se, 0.522/Sp, 0.99) (64).

For CD, FC showed the highest sensitivity: 0.95 (0.92–0.97) (21). FL showed the highest specificity: 1.00 (0.50–1.00) (44). Also, the specificity of anti-GP2 was good (49).

#### Adults

For IBD, only FC was performed with Se of 0.825 (0.661–0.920) and Sp of 0.900 (0.573–0.984) (5). For UC, there was a review showing that the Se and Sp for ANCA IgG were 0.67 (0.54–0.79) and 0.85 (0.70–0.94), respectively (39).

#### Children

For IBD, FC with a cutoff of 50 μg/g showed the highest AUC of 0.96 (21). The AUCs of other biomarkers [FC, CRP, ESR, PLT, Hb, and Alb (30)] ranged from 0.76 to 0.95. One review presented results of US from three primary studies: sensitivity range from 0.39 to 0.55 and specificity range from 0.90 to 1.00 (35).

For CD, FC with a cutoff of 100 μg/g performed best with a sensitivity of 1.00 (0.93–1.00) and specificity of 0.98 (0.93–1.00) (21). MRE (Se, 0.84; 0.77–0.90/Sp, 0.97; 0.91–0.99) (22) also performed well in SBCD.

### Diagnosis: Inflammatory Bowel Disease vs. Irritable Bowel Syndrome

#### Mixed Population

For IBD, FC with a cutoff of 50 μg/g was the most sensitive test with a sensitivity of 0.97 (0.91–0.99) (52). As for specificity, FL was the best: 0.94 (0.91–0.96) (24). One review presented the diagnostic performance of fecal S100A12 (Se, 0.86; 0.73–0.94/Sp, 0.96; 0.79–0.99) (39) (Supplementary Table 3).

### Diagnosis: Inflammatory Bowel Disease vs. Functional Gastrointestinal Disorders

#### Adults

For IBD, there was only one test: FC (Se, 0.88; 0.80–0.93/Sp, 0.71; 0.59–0.82) (53).

### Diagnosis: Crohn’s Disease vs. Ulcerative Colitis

#### Mixed Population

To differentiate CD from UC, the sensitivity of tests is generally low, including anti-GP2, ASCA (20, 54). ASCA IgA showed the highest specificity of 0.955 (0.938–0.967) (20). To differentiate UC from CD, the only test included in our analysis was ANCA (Se, 0.553; 0.530–0.576/Sp, 0.885; 0.871–0.898) (20).

### Diagnosis: Crohn’s Disease vs. Intestinal Tuberculosis

#### Mixed Population

IGRA (Se, 0.828; 0.784–0.855/Sp, 1.00; 0.867–0.896) (48) had better diagnostic performance than ASCA (Se, 0.828; 0.784–0.855/Sp, 0.867; 0.832–0.896) (25).

### Activity

#### Mixed Population

For IBD, FC with a cutoff of 50 μg/g presented the highest sensitivity of 0.92 (0.90–0.94) (42), and MRE showed the highest specificity of 0.93 (0.90–0.95) (30). Besides, other radiological examinations [US, leukocyte scintigraphy (LS), and CT] all performed well with balanced sensitivity and specificity (6, 30, 43, 56, 59). However, other biomarkers (CRP and FL) were not as good as radiological examinations (27). One review suggested a sensitivity range of 0.64 to 0.93 and a specificity range of 0.71 to 1, showing that the diagnostic accuracy of TAUS (transabdominal US) remains inconclusive (Supplementary Table 3) (35).

For CD, US showed the best specificity of 0.977 (0.700–0.999) (6). Contrast-enhanced ultrasound (CEUS) was the most sensitive test with a sensitivity of 0.94 (0.87–0.97) (45). CT and MRE also performed well; however, the sensitivity of DWI-MRE was poor (23, 28, 34, 47). FC and FL performed slightly worse than CT and MRE (32, 55). For UC, US had both best sensitivity (0.886, 0.800–0.939) and specificity (0.819, 0.456–0.961) (6). Among other tests, the specificity of FIT and FL and the sensitivity of FC and FL were fair (32, 36).

#### Adults

For IBD, US and CT have similar diagnostic performance. The sensitivity of ultrasound was slightly higher (0.860; 0.745–0.928) (6), while the specificity of CT was slightly higher (0.86; 0.81–0.90) (43). Monoclonal anti-granulocyte antibody scintigraphy (MAAS) was sensitive (Se, 0.94; 0.89–0.97), but its specificity was not good (Sp, 0.45; 0.37–0.53) (43).

#### Children

For IBD, US had great performance: Se, 0.876 (0.542–0.977); Sp, 1.00 (6). One review reported the diagnostic accuracy of TAUS, but showed that it remained inconclusive (Supplementary Table 3) (35). The other review showed that the sensitivity of positron emission tomography/CT (PET/CT): 0.59 (0.36–0.79) (SBFT, small-bowel follow through, used as the reference standard); 0.86 (0.70–0.95) (colonoscopy used as the reference standard) and the specificity: 1.00 (0.77–1.00) and 0.50 (0.01–0.99), respectively (43).

### Recurrence

#### Mixed Population

For IBD, the only test was FC (Se, 0.78; 0.72–0.83/Sp, 0.73; 0.68–0.77/AUC, 0.83) (40). For UC, the sensitivity and specificity of FC were 0.75 (0.70–0.79) and 0.77 (0.74–0.80), respectively (33). For CD, FC showed the sensitivity of 0.75 (0.64–0.84) and specificity of 0.71 (0.64–0.76) (40).

For postoperative CD, SICUS showed the highest sensitivity of 0.99 (0.99–1.00) (62). FC for clinical recurrence presented the highest specificity of 0.88 (0.80–0.93), while FC for endoscopic recurrence presented with better sensitivity (26). Besides, MRE and other subtypes of US performed well in both sensitivity and specificity (50, 51, 57).

### Reviews Eligible for Update

We searched for newly published studies for each moderate quality review (Supplementary Appendix 6). After screening, 8 reviews (20, 22, 26, 28–30, 32, 35) have eligible new published studies. However, after calculation, no reviews need to be updated. The overview of updating was presented in Supplementary Appendix 12.

## Discussion

Our detailed umbrella review synthesized existing systematic reviews and meta-analyses into one user-friendly document. A total of 106 associations, including 17 non-invasive tests, have been studied.

### Main Findings

Evidence from the umbrella review suggests that FC (0.99) and FL (0.82) were the most sensitive markers for distinguishing IBD from non-IBD. Similarly, ANCA (0.971) and FL (0.95) were the most specific marker for this purpose. To distinguish IBD from IBS, the most sensitive one was FC (cutoff 50 μg/g, 0.97; cutoff 100 μg/g, 0.92) and the most specific marker was FL (0.94). To distinguish CD from UC, all tests had low sensitivity, with ASCA IgA (0.955) having the highest specificity. IGRA (Se, 0.828; Sp, 0.867) was the best test to distinguish CD from ITB. There is only one test to diagnose IBD from FGID and only one test to distinguish UC from CD, FC, and ANCA. As for assessing activity, US (Se, 0.864; Sp, 0.883) and MRE (Se, 0.83; Sp, 0.93) perform well. The sensitivity of FC (0.85) was also good. As for postoperative recurrence of CD, SICUS (0.99) had the highest sensitivity and FC (CR: 0.88) had the highest specificity. We concluded that biomarkers played a good role in diagnosis, while radiological examinations, especially MRE and US, were more prominent in assessing activity and predicting recurrence. Supplementary Table 4 presents the characteristic of diagnostic performance and clinical use of each test.

### Strengths and Limitations

Compared with other studies summarizing non-invasive tests for IBD (65, 66), our umbrella review provides the first systematic appraisal of the evidence using robust criteria. We used the AMSTAR 2 tool to assess the quality of reviews and used CCA to evaluate the degree of overlapping and report the highest quality and most current review. Besides, our umbrella review included both blood, stool biomarkers and radiological examinations. Furthermore, we rigorously classified the assessments into age groups based on the exact age range of the primary studies included and into several groups to discuss the diagnostic performance in a different clinical condition more rigorously and reasonably.

Several limitations are present in this review. Lack of data, including missing meta-data, hindered the reporting of some elements of the umbrella review and lack of reviews of children or adults alone. In addition, one review (20) could not undergo the normal updating process because it did not report the included studies of each assessment. Besides, some reviews were rated as low quality for the most common reason: lack of protocol. However, registering protocol has been rare, especially in the IBD field. What’s more, since most articles do not report the value of AUC, we can’t do a good comparison and analysis of AUC.

### Implications for Practice and Future Research

This comprehensive umbrella review could help clinicians make better decisions about the appropriate tests prior to endoscopy. In terms of diagnosis, we suggested that in patients with symptoms suggestive of IBD in whom the clinician considers endoscopy, FC could be a sensitive test for safely excluding IBD. For patients with a negative result, we recommend that they continue to be monitored rather than do endoscopic examination immediately, unless it is very urgent. In patients with a positive result, FL is a good choice because of their low false-positive rate and consequent reduction of unnecessary endoscopies if patients are willing to have a stool test; if not, ANCA is an alternative. Clinicians can use our results to select a specific marker based on the practical situation. If both tests are positive, the patient is highly likely to have IBD. Endoscopic examination can be followed to confirm the diagnosis and disease classification. Radiation examinations, especially US and MRE, performed well in the activity assessment and predicting relapse. For patients with CD, we recommend having FC or US tests regularly to monitor the disease activity. Specifically, US or MRE is recommended for patients requiring postoperative recurrence monitoring. For patients with UC, MRE is the best choice to assess activity and predict relapse.

Our results show that there are not many reviews for children, especially in activity assessment and predicting recurrence. However, the use of endoscopy, invasive and requiring general anesthesia, can lead to child disobedience and disapproval of parents. An attitude of “wait and see” may cause unnecessary concerns and loss of wellbeing in children with IBD. Therefore, high-quality prospective studies on non-invasive testing in children should be complemented.

## Conclusion

In summary, this umbrella review summarized the diagnostic performance of non-invasive tests for IBD in different clinical conditions and age groups and offered our suggestions on how to use the non-invasive tests appropriately. Researchers and clinicians could choose a suitable test based on our results. Further studies on non-invasive tests in children are needed.

## Data Availability Statement

The original contributions presented in this study are included in the article/Supplementary Material, further inquiries can be directed to the corresponding authors.

## Author Contributions

All authors listed have made a substantial, direct, and intellectual contribution to the work, and approved it for publication.

## Author Disclaimer

The corresponding authors had full access to all the data in the study and has final responsibility for the decision to submit for publication.

## Conflict of Interest

The authors declare that the research was conducted in the absence of any commercial or financial relationships that could be construed as a potential conflict of interest.

## Publisher’s Note

All claims expressed in this article are solely those of the authors and do not necessarily represent those of their affiliated organizations, or those of the publisher, the editors and the reviewers. Any product that may be evaluated in this article, or claim that may be made by its manufacturer, is not guaranteed or endorsed by the publisher.
