# Supplementary material for: Diagnostic Utility of Non-invasive Tests for Inflammatory Bowel Disease: An Umbrella Review
Source: Front Med (Lausanne). 2022 Jul 11;9:920732. doi: 10.3389/fmed.2022.920732 (PMC9337241; doi:10.3389/fmed.2022.920732)
Supplement: Supplementary file 1 [file Data_Sheet_1.docx]

**Supplemental materials**

**Appendix of contents**

**Appendix 1:** Search Strategy for systematic review

**Appendix 2:** process of identifying age groups

**Appendix 3:** Citation matrices for reviews with overlapping assessments

**Appendix 4:** management of overlapping reviews

**Appendix 5:** Search strategy for newly published studies

**Appendix 6:** Evaluation process for considering reviews for update

**Appendix 7:** Study selection

**Appendix 8:** List of excluded studies

**Appendix 9:** AMSTAR 2 quality appraisal scores

**Appendix 10:** General characteristics of reviews with overlapping associations

**Appendix 11:** A: List of studies with non-overlapping associations included in umbrella review Analysis

B: List of contemporary overlapping studies excluded from review

**Appendix 12:** Results of updating: meta-analysis

**Appendix 13:** Overview of updating

**Table of contents**

**Table S1:** General characteristics of systematic reviews included in the umbrella

review

**Table S2:** Tabular presentation of findings: Meta-analysis

**Table S3:** Tabular presentation of findings: Narrative syntheses

**Table S4:** General characteristic in diagnostic performance and clinical use of each tests.

**Appendix 1:** Search Strategy for systematic review

| **Database** | **Search strategy** |
| --- | --- |
| Pubmed | ("Sensitivity and Specificity"[Mesh] OR specificit*[tw] OR false negative[tw] OR accura*[tw] OR sensitiv*[tw] OR “reproducibility of results”[MeSH]) AND ("Inflammatory bowel diseases"[MeSH] OR inflammatory bowel disease*[tw] OR IBD[tw] OR Colitis [tw] OR Crohn [tw]) |
| Embase | ('enteritis'/de OR 'colitis'/exp OR 'inflammatory bowel disease':de,ab,ti OR IBD:de,ab,ti OR colitis:de,ab,ti OR crohn:de,ab,ti) AND ('sensitivity and specificity'/exp OR 'predictive value'/exp OR 'receiver operating characteristic'/exp OR sensitiv*:de,ab,ti OR specificit*:de,ab,ti OR accura*:de,ab,ti OR 'false negative':de,ab,ti OR ‘reproducibility’/exp) NOT [medline]/lim AND [embase]/lim AND ([cochrane review]/lim OR [systematic review]/lim OR [meta analysis]/lim) |
| Cochrane | #1 MeSH descriptor: [Inflammatory Bowel Diseases] explode all trees  #2 inflammatory bowel disease* or IBD or Colitis or Crohn  #3 #1 or #2  #4 MeSH descriptor: [Sensitivity and Specificity] explode all trees  #5 specificit* or false negative or accura* or sensitive*  #6 MeSH descriptor: [Reproducibility of Results] explode all trees  #7 #4 or #5 or #6  #8 #3 or #7  #9 systematic review or meta  #10 #8 and #9 |
| Web of Science | #1 TS=(Inflammatory bowel diseases) OR ALL=(inflammatory bowel disease* OR IBD OR Colitis OR Crohn)  #2 TS=(Sensitivity and Specificity OR reproducibility of results) OR ALL=(specificit* OR false negative OR accura* OR sensitiv*)  #3 TS= (systematic review OR meta-analysis)  #4 #1 AND #2 AND #3 |

**Appendix 2:** process of identifying age groups

Age ranges stated in a review

Age ranges not stated in a review

extract the age range of primary studies by full-text screening

primary studies all provide an age range

primary studies do not provide an age range

Sent an email to author for inquiry

Receive an answer from author

Not answered

Identifying age groups

<18 years

>18 years

Both <18 years and >18 years included

Children

Adults

Mixed population

**Appendix 3:** Citation matrices for reviews with overlapping assessments

**A. Diagnosis**

**A1. Accuracy of FC in IBD (mixed)**

| **systematic review** | holtman2015 | Petryszyn2019 |
| --- | --- | --- |
| **primary study** |  |  |
| Ashorn2009 | ✔ |  |
| Bonnín2007 | ✔ |  |
| Banerjee2015 |  | ✔ |
| Canani2006 | ✔ |  |
| Caviglia2014 |  | ✔ |
| Conroy2018 |  | ✔ |
| Carroccio2003 |  | ✔ |
| Diamanti2010 | ✔ |  |
| Damms2008 |  | ✔ |
| D’Incà2007 |  | ✔ |
| Fagerberg2005 | ✔ |  |
| Sidler2008 | ✔ |  |
| Van de Vijver2012 | ✔ |  |
| Henderson2012 | ✔ |  |
| Minar2014 | ✔ |  |
| Mowat2016 |  | ✔ |
| Perminow2009 | ✔ |  |
| Pavlidis2013 |  | ✔ |
| Walker2018 |  | ✔ |
| Kalantari2015 |  | ✔ |
| Kennedy2015 |  | ✔ |
| Kok2012 |  | ✔ |
| Oyaert2014 |  | ✔ |
| Otten2008 |  | ✔ |
| Tibble2000 |  | ✔ |
| Limburg2000 |  | ✔ |
| Schröder2007 |  | ✔ |
| Schoepfer2008 |  | ✔ |
| Schoepfer2007 |  | ✔ |
| **Total** (No of publications per review) | **10** | **19** |
| **Grand Total (N)** | **29** | |
| **Rows (r)** | **29** | |
| **Columns (c)** | **2** | |
| **CCA** | **0.00%** | |

**A2 Accuracy of anti-GP2 in CD (mixed)**

| **systematic review** | Deng2017 | Gkiouras2020 |
| --- | --- | --- |
| **primary study** |  |  |
| Roggenbuck2011 | ✔ |  |
| Pavlidis2016 | ✔ |  |
| Michaels2015 | ✔ | ✔ |
| Roggenbuck2015 | ✔ | ✔ |
| Zhang2015 | ✔ |  |
| Pavlidis2015 | ✔ |  |
| Laass2015 | ✔ |  |
| Papp2015 | ✔ | ✔ |
| Bogdanos2012 | ✔ | ✔ |
| Op De Beéck2012 | ✔ |  |
| Pavlidis2016 |  | ✔ |
| **Total** (No of publications per review) | **10** | **5** |
| **Grand Total (N)** | **15** | |
| **Rows (r)** | **11** | |
| **Columns (c)** | **2** | |
| **CCA** | **36.36%** | |

**A3. Accuracy of anti-GP2 IgA in CD (mixed)**

| **systematic review** | Deng C2017 | Gkiouras2020 |
| --- | --- | --- |
| **primary study** |  |  |
| Roggenbuck2011 | ✔ |  |
| Pavlidis2016 | ✔ | ✔ |
| Michaels2015 | ✔ | ✔ |
| Roggenbuck2015 | ✔ |  |
| Bonaci-Nikolic2012 | ✔ | ✔ |
| Bogdanos2012 | ✔ |  |
| Op De Beéck2012 | ✔ | ✔ |
| Pavlidis2011 | ✔ |  |
| Roggenbuck2009 | ✔ | ✔ |
| Papp2015 |  | ✔ |
| Gross2014 |  | ✔ |
| Roggenbuck2011 |  | ✔ |
| Caneparo2016 |  | ✔ |
| Pavlidis2015 |  | ✔ |
| Bogdanos2012 |  | ✔ |
| Roggenbuck2015 |  | ✔ |
| Zhang2018 |  | ✔ |
| Zhang2015 |  | ✔ |
| **Total** (No of publications per review) | **9** | **14** |
| **Grand Total (N)** | **23** | |
| **Rows (r)** | **18** | |
| **Columns (c)** | **2** | |
| **CCA** | **27.78%** | |

**A4. Accuracy of anti-GP2 IgG in CD (mixed)**

| **systematic review** | Deng C2017 | Gkiouras2020 |
| --- | --- | --- |
| **primary study** |  |  |
| Roggenbuck2011 | ✔ | ✔ |
| Pavlidis2016 | ✔ | ✔ |
| Michaels2015 | ✔ | ✔ |
| Roggenbuck2015 | ✔ | ✔ |
| Zhang2015 | ✔ | ✔ |
| Pavlidis2015 | ✔ | ✔ |
| Laass2015 | ✔ |  |
| Papp2015 | ✔ | ✔ |
| Kohoutova2014 | ✔ |  |
| Gross2014 | ✔ | ✔ |
| Komorowski2013 | ✔ |  |
| Bonaci-Nikolic2012 | ✔ | ✔ |
| Pavlidis2012 | ✔ | ✔ |
| Bogdanos2012 | ✔ | ✔ |
| Op De Beéck2012 | ✔ | ✔ |
| Pavlidis2011 | ✔ |  |
| Roggenbuck2009 | ✔ | ✔ |
| Caneparo2016 |  | ✔ |
| Zhang2018 |  | ✔ |
| **Total** (No of publications per review) | **17** | **15** |
| **Grand Total (N)** | **32** | |
| **Rows (r)** | **19** | |
| **Columns (c)** | **2** | |
| **CCA** | **68.42%** | |

**B. Activity**

**B1. Accuracy of FC in UC (mixed)**

| **systematic review** | Rokkas2018 | Ye2021 |
| --- | --- | --- |
| **primary study** |  |  |
| Schoepfer2009 | ✔ |  |
| Onal 2012 | ✔ |  |
| Schoepfer2013 | ✔ |  |
| Lobaton2013 | ✔ |  |
| Hosseini 2015 | ✔ |  |
| Kristensen2015 | ✔ |  |
| Kawashima2020 |  | ✔ |
| Magro2020 |  | ✔ |
| Sagami2020 |  | ✔ |
| Shi2017 |  | ✔ |
| **Total** (No of publications per review) | **6** | **4** |
| **Grand Total (N)** | **10** | |
| **Rows (r)** | **10** | |
| **Columns (c)** | **2** | |
| **CCA** | **0.00%** | |

**B2. Accuracy of FIT in UC (mixed)**

| **systematic review** | Dai2018 | Lee2020 |
| --- | --- | --- |
| **primary study** |  |  |
| Nakarai2013 | ✔ | ✔ |
| Takashima2015 | ✔ | ✔ |
| Shi2017 | ✔ |  |
| Ma2017 | ✔ |  |
| Hiraoka2017 | ✔ | ✔ |
| Ryu2016 | ✔ | ✔ |
| Nakarai2016 | ✔ | ✔ |
| Nakarai2018 |  | ✔ |
| **Total** (No of publications per review) | **7** | **6** |
| **Grand Total (N)** | **13** | |
| **Rows (r)** | **8** | |
| **Columns (c)** | **2** | |
| **CCA** | **62.50%** | |

**B3. Accuracy of SICUS in SBCD (mixed)**

| **systematic review** | Zhu2016 | Kopylov2017 |
| --- | --- | --- |
| **primary study** |  |  |
| Calabrese2013 | ✔ |  |
| Pallotta2013 | ✔ |  |
| **Pallotta2012** | ✔ |  |
| **Pallotta2005** | ✔ |  |
| **Onali2012** | ✔ |  |
| **Kumar2015** | ✔ |  |
| Aloi2015 | ✔ | ✔ |
| Chatu2012 | ✔ |  |
| **Parente2004** | ✔ |  |
| **Calabrese2009** | ✔ |  |
| Cittadini2001 | ✔ |  |
| Biancone2007 | ✔ | ✔ |
| Castiglione2008 | ✔ |  |
| Oliva2016 |  | ✔ |
| Petruzziello2011 |  | ✔ |
| Petruzziello2010 |  | ✔ |
| **Total** (No of publications per review) | **13** | **5** |
| **Grand Total (N)** | **18** | |
| **Rows (r)** | **16** | |
| **Columns (c)** | **2** | |
| **CCA** | **12.50%** | |

**B4. Accuracy of CT in CD (mixed)**

| **systematic review** | Panés2011 | Qiu2014 |
| --- | --- | --- |
| **primary study** |  |  |
| Fiorino2011 | ✔ |  |
| Hassan2003 | ✔ |  |
| Lee2009 | ✔ | ✔ |
| Low2000 | ✔ |  |
| Fiorino2011 |  | ✔ |
| Jensen, Kjeldsen2011 |  | ✔ |
| Jensen, Nathan2011 |  | ✔ |
| Schmidt2010 |  | ✔ |
| Siddiki2009 |  | ✔ |
| **Total** (No of publications per review) | **4** | **6** |
| **Grand Total (N)** | **10** | |
| **Rows (r)** | **9** | |
| **Columns (c)** | **2** | |
| **CCA** | **11.11%** | |

**B5. Accuracy of FC (cut-off 50) in SBCD (mixed)**

| **systematic review** | Kopylov2016 | Jung2020 |
| --- | --- | --- |
| **primary study** |  |  |
| Egea-Valenzuela2015 | ✔ | ✔ |
| Jensen2011 | ✔ | ✔ |
| Kopylov, Nemeth2015 | ✔ | ✔ |
| Kopylov, Yablecovitch2015 | ✔ | ✔ |
| Koulaouzidis2011 | ✔ | ✔ |
| Olsen2015 | ✔ | ✔ |
| Sipponen2012 | ✔ | ✔ |
| Hale2016 |  | ✔ |
| Monteiro2018 |  | ✔ |
| Yousuf22018 |  | ✔ |
| **Total** (No of publications per review) | **7** | **10** |
| **Grand Total (N)** | **17** | |
| **Rows (r)** | **10** | |
| **Columns (c)** | **2** | |
| **CCA** | **70.00%** | |

**B6. Accuracy of FC (cut-off 100) in SBCD (mixed)**

| **systematic review** | Kopylov2016 | Jung2020 |
| --- | --- | --- |
| **primary study** |  |  |
| Egea-Valenzuela2015 | ✔ | ✔ |
| Kopylov, Nemeth2015 | ✔ | ✔ |
| Kopylov, Yablecovitch2015 | ✔ | ✔ |
| Koulaouzidis2011 | ✔ | ✔ |
| Sipponen2012 | ✔ | ✔ |
| Aggarwal2017 |  | ✔ |
| Bar-Gil2017 |  | ✔ |
| Egea Valenzuela2016 |  | ✔ |
| Hale2016 |  | ✔ |
| Kopylov2018 |  | ✔ |
| Monteiro2018 |  | ✔ |
| Yousuf2018 |  | ✔ |
| **Total** (No of publications per review) | **5** | **12** |
| **Grand Total (N)** | **17** | |
| **Rows (r)** | **12** | |
| **Columns (c)** | **2** | |
| **CCA** | **41.67%** | |

**B7. Accuracy of FC (cut-off 200) in SBCD (mixed)**

| **systematic review** | Kopylov2016 | Jung2020 |
| --- | --- | --- |
| **primary study** |  |  |
| Kopylov, Nemeth2015 | ✔ | ✔ |
| Kopylov, Yablecovitch2015 | ✔ | ✔ |
| Koulaouzidis2011 | ✔ | ✔ |
| Sipponen2012 | ✔ | ✔ |
| Hale2016 |  | ✔ |
| Monteiro2018 |  | ✔ |
| Yousuf2018 |  | ✔ |
| **Total** (No of publications per review) | **4** | **7** |
| **Grand Total (N)** | **11** | |
| **Rows (r)** | **7** | |
| **Columns (c)** | **2** | |
| **CCA** | **57.14%** | |

**B8. Accuracy of US in IBD (mixed)**

| **systematic review** | Sagami2020 | Alshammari2021 |
| --- | --- | --- |
| **primary study** |  |  |
| Pascu2004 | ✔ | ✔ |
| Jesus Martinez2019 |  | ✔ |
| Vigano2019 |  | ✔ |
| Yuksel2019 |  | ✔ |
| Faure1997 | ✔ |  |
| Maconi1996 | ✔ |  |
| Futagami1999 | ✔ |  |
| Neye2003 | ✔ |  |
| Neye2004 | ✔ |  |
| Parente2008 | ✔ |  |
| Moreno2014 | ✔ |  |
| Civitelli2014 | ✔ |  |
| Dilillo2014 | ✔ |  |
| Shimazaki2016 | ✔ |  |
| Novak2017 | ✔ |  |
| Shiraki2017 | ✔ |  |
| Pradeep2018 | ✔ |  |
| Hashimoto2018 | ✔ |  |
| Kinoshita2019 | ✔ |  |
| Allocca2018 | ✔ |  |
| Allocca2018-2 | ✔ |  |
| Sagami2019 | ✔ |  |
| **Total** (No of publications per review) | **19** | **4** |
| **Grand Total (N)** | **23** | |
| **Rows (r)** | **22** | |
| **Columns (c)** | **2** | |
| **CCA** | **4.55%** | |

**B9. Accuracy of MRI in SBCD (mixed)**

| **systematic review** | Qiu2014 | Ahmad2021 |
| --- | --- | --- |
| **primary study** |  |  |
| Fiorino2011 | ✔ |  |
| Jensen,Kjeldsen2011 | ✔ |  |
| Jensen,Nathan2011 | ✔ |  |
| Lee2009 | ✔ |  |
| Schmidt2010 | ✔ |  |
| Siddiki2009 | ✔ |  |
| Yoon2015 |  | ✔ |
| Bettenworth2016 |  | ✔ |
| Sinha2011 |  | ✔ |
| Rimola2017- J Gastroenterol 52:585–593 |  | ✔ |
| Rimola2017- Abdom Radiol (NY) 42:2783–2791. |  | ✔ |
| Tielbeek2013 |  | ✔ |
| Ordás2019 |  | ✔ |
| Pomerri2017 |  | ✔ |
| **Total** (No of publications per review) | **6** | **8** |
| **Grand Total (N)** | **14** | |
| **Rows (r)** | **14** | |
| **Columns (c)** | **2** | |
| **CCA** | **0.00%** | |

**C. Recurrence**

**C1. Accuracy of US in postoperative CD (mixed)**

| **systematic review** | Greenup2016 | Yung2017 | Rispo2018 |
| --- | --- | --- | --- |
| **primary study** |  |  |  |
| Calabrese2009 | ✔ | ✔ | ✔ |
| Castiglione2008 | ✔ | ✔ | ✔ |
| Pallotta2010 | ✔ |  | ✔ |
| Paredes2010 | ✔ |  | ✔ |
| Paredes2013 | ✔ | ✔ | ✔ |
| Rispo2006 | ✔ | ✔ | ✔ |
| Andreoli2013 |  | ✔ |  |
| Onali2010 |  | ✔ | ✔ |
| Andreoli1998 |  |  | ✔ |
| Biancone2007 |  |  | ✔ |
| Onali2016 |  |  | ✔ |
| **Total** (No of publications per review) | **6** | **6** | **10** |
| **Grand Total (N)** | **22** | | |
| **Rows (r)** | **11** | | |
| **Columns (c)** | **3** | | |
| **CCA** | **50.00%** | | |

**C1. Accuracy of MRI in postoperative CD (mixed)**

| **systematic review** | Panés2011 | Yung2017 |
| --- | --- | --- |
| **primary study** |  |  |
| Sailer2008 | ✔ | ✔ |
| Gallego2011 |  | ✔ |
| Aguas2012 |  | ✔ |
| Miao2002 | ✔ |  |
| Koh2001 | ✔ |  |
| Florie2005 | ✔ |  |
| Girometti2008 | ✔ |  |
| **Total** (No of publications per review) | **5** | **3** |
| **Grand Total (N)** | **8** | |
| **Rows (r)** | **7** | |
| **Columns (c)** | **2** | |
| **CCA** | **14.29%** | |

Formula for calculating the corrected covered area, **CCA (%) = N-r / rc-r**

Where N = number of included publications (sum of checked boxes), r = number of rows (primary publications), c = number of columns (number of reviews).

**Appendix 4:** Management of overlapping reviews

Overlapping reviews

Outdated review: excluded

Remaining reviews

Calculate their degree of overlap (CCA)

Cochrane vs non-Cochrane

Choose: Cochrane

non-Cochrane vs non-Cochrane

CCA≥11%

CCA≤10%

preference was given to the review that: had the highest rating assessed with the AMSTAR 2 quality assessment tool; had the highest number of studies or participants; was most recent; supplied pooled effect estimates or had conducted a meta-analysis

both reviews were retained and the findings compared

**Appendix 5:** Search strategy for newly published studies

1. Pubmed search strategy for accuracy of ASCA and ANCA in IBD (update for Reese2006)

| **Search number** | **Search terms** | **Results** |
| --- | --- | --- |
| 1 | "antibodies, antineutrophil cytoplasmic"[MeSH Terms] OR ("antibodies"[All Fields] AND "antineutrophil"[All Fields] AND "cytoplasmic"[All Fields]) OR "antineutrophil cytoplasmic antibodies"[All Fields] OR ("anti"[All Fields] AND "neutrophil"[All Fields] AND "cytoplasmic"[All Fields] AND "antibodies"[All Fields]) OR "anti neutrophil cytoplasmic antibodies"[All Fields] OR ("panca"[All Fields] OR "pancas"[All Fields]) OR ("Anti-Saccharomyces"[All Fields] AND ("cerevisia"[All Fields] OR "cerevisiae"[All Fields] OR "cerevisiae s"[All Fields] OR "cerevisiaes"[All Fields]) AND ("antibodie"[All Fields] OR "antibodies"[MeSH Terms] OR "antibodies"[All Fields] OR "antibody s"[All Fields] OR "antibodys"[All Fields] OR "immunoglobulins"[MeSH Terms] OR "immunoglobulins"[All Fields] OR "antibody"[All Fields])) OR "ASCA"[All Fields] | 8291 |
| 2 | "inflammatory bowel diseases"[MeSH Terms] OR ("inflammatory"[All Fields] AND "bowel"[All Fields] AND "diseases"[All Fields]) OR "inflammatory bowel diseases"[All Fields] OR ("inflammatory"[All Fields] AND "bowel"[All Fields] AND "disease"[All Fields]) OR "inflammatory bowel disease"[All Fields] OR ("crohn disease"[MeSH Terms] OR ("crohn"[All Fields] AND "disease"[All Fields]) OR "crohn disease"[All Fields] OR "crohn s disease"[All Fields]) OR ("colitis, ulcerative"[MeSH Terms] OR ("colitis"[All Fields] AND "ulcerative"[All Fields]) OR "ulcerative colitis"[All Fields] OR ("ulcerative"[All Fields] AND "colitis"[All Fields])) | 124314 |
| 3 | #1 AND #2 | 984 |
| 4 | ("2006"[Date - Publication] : "3000"[Date - Publication]) | 15835371 |
| 5 | #3 AND #4 | 506 |

2. Pubmed search strategy for accuracy of MRE in paediatric IBD (update for Giles2007)

| **Search number** | **Search terms** | **Results** |
| --- | --- | --- |
| 1 | "inflammatory bowel diseases"[MeSH Terms] | 85602 |
| 2 | "magnetic resonance imaging"[MeSH Terms] OR ("magnetic"[All Fields] AND "resonance"[All Fields] AND "imaging"[All Fields]) OR "magnetic resonance imaging"[All Fields] | 600470 |
| 3 | ("intestine, small"[MeSH Terms] OR ("intestine"[All Fields] AND "small"[All Fields]) OR "small intestine"[All Fields] OR ("small"[All Fields] AND "bowel"[All Fields]) OR "small bowel"[All Fields]) AND ("image"[All Fields] OR "image s"[All Fields] OR "imaged"[All Fields] OR "imager"[All Fields] OR "imager s"[All Fields] OR "imagers"[All Fields] OR "images"[All Fields] OR "imaging"[All Fields] OR "imaging s"[All Fields] OR "imagings"[All Fields]) | 25179 |
| 4 | #1 AND #2 AND #3 | 629 |
| 5 | ("2013"[Date - Publication] : "3000"[Date - Publication]) AND #4 | 346 |
| 6 | ((#5 ) NOT (review[Publication Type])) NOT (meta-analysis[Publication Type]) | 258 |

3. Pubmed search strategy for accuracy of FC in assessing postoperative recurrence in CD (update for Qiu2015)

| **Search number** | **Search terms** | **Results** |
| --- | --- | --- |
| 1 | (calprotectin[MeSH Terms]) AND (Crohn’s disease[MeSH Terms]) | 445 |
| 2 | (calprotectin[MeSH Terms]) AND (IBD[Text Word]) | 459 |
| 3 | (Leukocyte L1 complex[MeSH Terms]) AND (IBD[Text Word]) | 459 |
| 4 | (calprotectin[MeSH Terms]) AND (relapse[MeSH Terms]) | 158 |
| 5 | (calprotectin[MeSH Terms]) AND (intestinal inflammation[MeSH Terms]) | 105 |
| 6 | #1 OR #2 OR #3 OR #4 OR #5 | 857 |
| 7 | (("2014"[Date - Publication] : "3000"[Date - Publication])) AND (#7) | 605 |
| 8 | (((#8 ) NOT (review[Publication Type])) NOT (systematic review[Publication Type])) NOT (meta-analysis[Publication Type]) | 537 |

4. Medline search strategy for accuracy of MR in assessing activity in SBCD (update for Ahmed2016)

| **Search number** | **Search terms** | **Results** |
| --- | --- | --- |
| 1 | exp Inflammatory Bowel Diseases/ | 85574 |
| 2 | Bowel Diseases, Inflammatory.af. | 12 |
| 3 | exp Crohn Disease/ | 40472 |
| 4 | exp Colitis, Ulcerative/ | 36189 |
| 5 | crohn$.mp. | 59748 |
| 6 | exp Colitis/ | 55659 |
| 7 | exp Ileitis/ | 3603 |
| 8 | (ulcer$ adj colit$).mp. | 43091 |
| 9 | 1 or 2 or 3 or 4 or 5 or 6 or 7 or 8 | 120795 |
| 10 | Magnetic Resonance Imaging.af. | 557680 |
| 11 | Imaging, Magnetic Resonance.af. | 888 |
| 12 | NMR Imaging.af. | 1358 |
| 13 | Imaging, NMR.af. | 51 |
| 14 | Zeugmatography.af. | 22 |
| 15 | Tomography, MR.af. | 133 |
| 16 | Tomography, NMR.af. | 21 |
| 17 | MR Tomography.af. | 362 |
| 18 | NMR Tomography.af. | 174 |
| 19 | Proton Spin Tomography.af. | 34 |
| 20 | Magnetization Transfer Contrast Imaging.af. | 44 |
| 21 | MRI Scans.af. | 10953 |
| 22 | MRI Scan.af. | 5215 |
| 23 | Scan, MRI.af. | 444 |
| 24 | Scans, MRI.af. | 119 |
| 25 | fMRI.af. | 49494 |
| 26 | MRI, Functional.af. | 230 |
| 27 | Functional MRI.af. | 13612 |
| 28 | Functional MRIs.af. | 21 |
| 29 | Magnetic Resonance Imaging, Functional.af. | 85 |
| 30 | Imaging, Chemical Shift.af. | 19 |
| 31 | Chemical Shift Imaging.af. | 1053 |
| 32 | 10 or 11 or 12 or 13 or 14 or 15 or 16 or 17 or 18 or 19 or 20 or 21 or 22 or 23 or 24 or 25 or 26 or 27 or 28 or 29 or 30 or 31 | 571382 |
| 33 | 9 and 32 | 2180 |
| 34 | limit 33 to yr="2016 -Current" | 806 |
| 35 | limit 34 to (comment or editorial or interview or letter) | 31 |
| 36 | 34 not 35 | 775 |

5. Pubmed search strategy for accuracy of MRE in assessing activity in paediatric IBD (update for Kopylov2016)

| **Search number** | **Search terms** | **Results** |
| --- | --- | --- |
| 1 | (capsule endoscopy[MeSH Terms]) AND (calprotectin[MeSH Terms]) | 41 |
| 2 | (("2016"[Date - Publication] : "3000"[Date - Publication])) AND (#1) | 24 |

6. Pubmed search strategy for accuracy of MRE in assessing active inflammation in paediatric IBD (update for Yoon2017)

| **Search number** | **Search terms** | **Results** |
| --- | --- | --- |
| 1 | (((child*[Text Word]) OR (pediatric*[Text Word])) OR (pediatric*[Text Word])) OR (adolescent*[Text Word]) | 3737089 |
| 2 | ((Crohn*[Text Word]) OR (inflammatory bowel disease[MeSH Terms])) OR (IBD[Text Word]) | 105923 |
| 3 | (((magnetic resonance enterography[Text Word]) OR (MR enterography[Text Word])) OR (MRE[Text Word])) OR (MR-E[Text Word]) | 2897 |
| 4 | #1 AND #2 AND #3 | 309 |
| 5 | (#4) AND (("2017"[Date - Publication] : "3000"[Date - Publication])) | 137 |
| 6 | (((#5) NOT (review[Publication Type])) NOT (systematic review[Publication Type])) NOT (meta-analysis[Publication Type]) | 114 |

7. Pubmed search strategy for accuracy of FC in assessing activity in IBD (update for Rokkas2018)

| **Search number** | **Search terms** | **Results** |
| --- | --- | --- |
| 1 | („calprotectin"[All Fields]) AND („faeces"[All Fields] OR „feces"[MeSH Terms] OR „feces"[All Fields]) | 1370 |
| 2 | („Crohn disease"[MeSH Terms] OR („Crohn"[All Fields] AND „disease"[All Fields]) OR „Crohn disease"[All Fields] OR („Crohn‘s"[All Fields] AND „disease"[All Fields]) OR „Crohn‘s disease"[All Fields]) | 59572 |
| 3 | („colitis, ulcerative"[MeSH Terms]) OR („colitis"[All Fields] AND „ulcerative"[All Fields]) OR „ulcerative colitis"[All Fields] OR („ulcerative"[All Fields] AND „colitis"[All Fields]) | 52256 |
| 4 | #1 AND #2 AND #3 | 291 |
| 5 | (#4) AND (("2018"[Date - Publication] : "3000"[Date - Publication])) | 87 |

8. Pubmed search strategy for accuracy of TAUS in assessing activity in paediatric IBD (update for van Wassenaer2019)

| **Search number** | **Search terms** | **Results** |
| --- | --- | --- |
| 1 | ("Inflammatory Bowel Diseases"[Mesh] OR "Proctitis"[Mesh] OR inflammatory bowel disease*[tiab] OR crohn*[tiab] OR colitis[tiab] OR IBD[tiab] OR proctitis[tiab] OR Ileitis[tiab]) AND ("Ultrasonography"[Mesh] OR ultraso*[tiab] OR echo*[tiab] OR sono*[tiab]) AND ("Colonoscopy"[Mesh] OR "Endoscopy"[Mesh] OR MRE[tiab] OR MR[tiab] OR magnetic resonance entero*[tiab] OR MaRia[tiab] OR Clermont score*[tiab] OR London score*[tiab] OR colonoscop*[tiab] OR ileocolonoscop*[tiab] OR endoscop*[tiab] OR Mayo[tiab] OR SES-CD[tiab] OR CDEIS[tiab] OR Rutgeerts[tiab]) AND ("Pediatrics"[Mesh] OR "Child"[Mesh] OR "Adolescent"[Mesh] OR child*[tiab] OR adolescen*[tiab] OR teen*[tiab] OR youth[tiab] OR young[tiab] OR pediatric*[tiab] OR paediatric*[tiab]) AND ("Sensitivity and Specificity"[Mesh] OR "Diagnostic Errors"[Mesh] OR sensitiv*[tiab] OR specific*[tiab] OR correl*[tiab] OR accura*[tiab] OR golden standard[tiab] OR gold standard[tiab] OR (reference[tiab] AND (test[tiab] OR standard[tiab])) OR index test[tiab] OR valid*[tiab] OR "Validation Studies" [Publication Type] OR verif*[ti] OR "Evaluation Studies" [Publication Type] OR evaluat*[ti] OR (false[tiab] AND (positive[tiab] OR negative[tiab])) OR pretest[tiab] OR pre-test[tiab] OR posttest[tiab] OR post-test[tiab] OR predictive value*[tiab] OR predict*[ti] OR roc[tiab] OR likelyhood[tiab] OR likelihood[tiab] OR value*[ti] OR "Reference Values"[Mesh] OR cutoff[tiab] OR cut-off[tiab] OR "Quality Control"[Mesh] OR "Reproducibility of Results"[Mesh] OR repeatability[tiab] OR reproducibility[tiab] OR efficacy[tiab] OR reliability[tiab] OR "Comparative Study" [Publication Type] OR odds[tiab] OR error*[tiab] OR suitability[tiab] OR utility[tiab]) | 220 |
| 2 | (#1 ) AND (("2019"[Date - Publication] : "3000"[Date - Publication])) | 31 |

**Appendix 6:** Evaluation process for considering reviews for update

Original Systematic Review

Reviews without a meta-analysis: discuss with senior authors

Meta-analysis: if the addition of newly published studies resulted in a change of statistical significance or a change in the result by at least 50%

Conducting update search using the original SR search strategy

Exporting the identified articles to a reference manager datababase

Screening title and abstract

Number of included studies

Number of excluded studies

Screening full text

Number of included studies

Number of excluded studies

Extracting data

Identifying qualitative and/or quantitative signals updating

Deciding on updating status of SR

Low priority for updating

Medium priority for updating

Low priority for updating

**Appendix 7:** Study selection

**
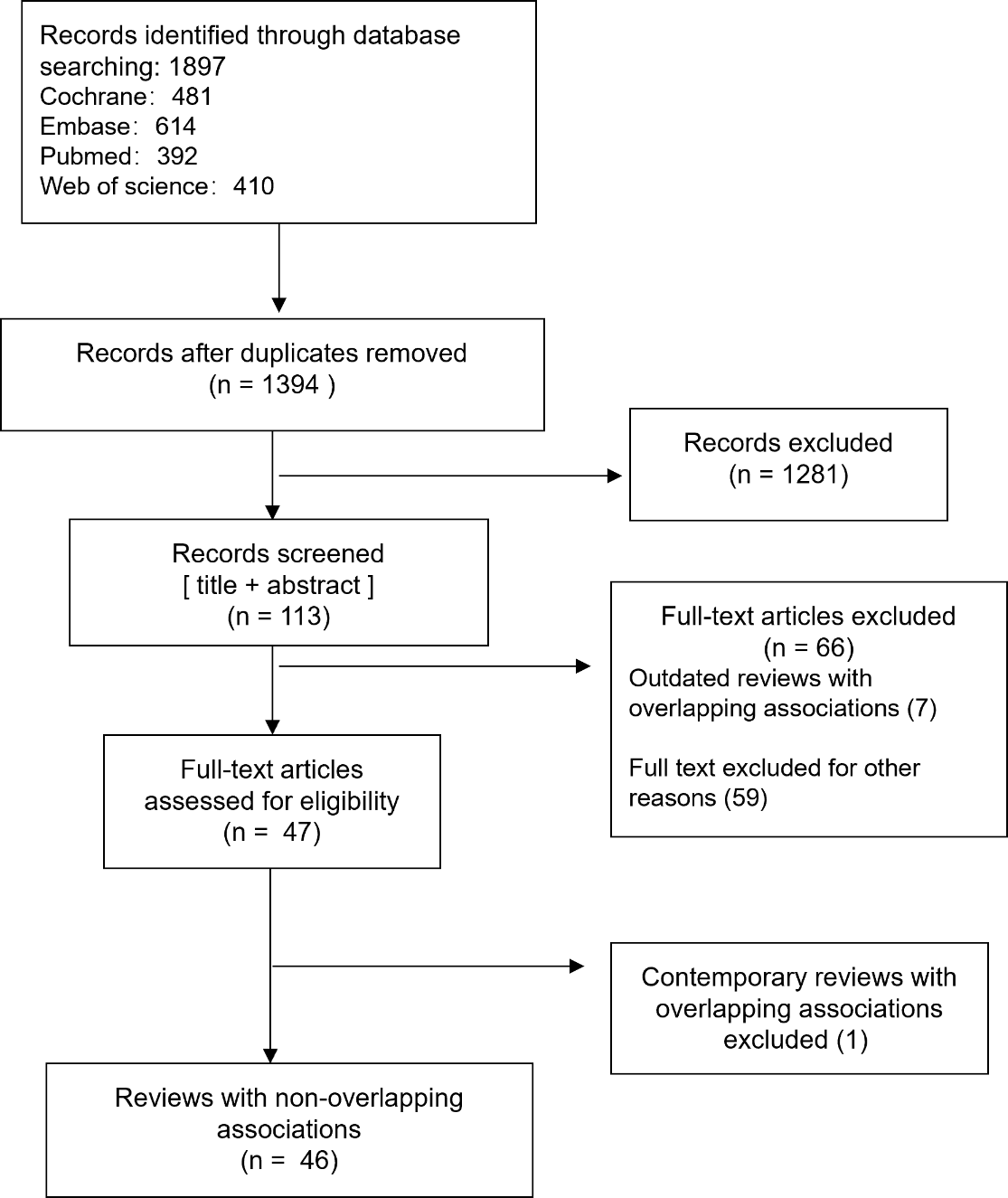
**

**Appendix 8:** List of excluded studies

|  | **Author** | **Year** | **Title** | **Reason for Exclusion** |
| --- | --- | --- | --- | --- |
| 1 | Michael R Konikoff | 2006 | Role of fecal calprotectin as a biomarker of intestinal inflammation in inflammatory bowel disease | No Quality appraisal of primary studies |
| 2 | Bonneau J | 2014 | Systematic review: new serological markers (anti-glycan, anti-GP2, anti-GM-CSF Ab) in the prediction of IBD patient outcomes | No Quality appraisal of primary studies |
| 3 | Bannaga AS | 2019 | Diagnosing Inflammatory bowel disease using noninvasive applications of volatile organic compounds: a systematic review | No Quality appraisal of primary studies |
| 4 | Vernia F | 2019 | Is fecal calprotectin an accurate marker in the management of Crohn's disease? | No Quality appraisal of primary studies |
| 5 | Luo Y | 2020 | Molecular Magnetic Resonance Imaging with Contrast Agents for Assessment of Inflammatory Bowel Disease: A Systematic Review | No Quality appraisal of primary studies |
| 6 | Smith RL | 2020 | Systematic Review: Clinical Utility of Gastrointestinal Ultrasound in the Diagnosis, Assessment and Management of Patients With Ulcerative Colitis | No Quality appraisal of primary studies |
| 7 | Nijakowski K | 2020 | Salivary Biomarkers for Diagnosis of Inflammatory Bowel Diseases: A Systematic Review | No Quality appraisal of primary studies |
| 8 | Gole | 2019 | Pre-Treatment Biomarkers of Anti-Tumour Necrosis Factor Therapy Response in Crohn's Disease-A Systematic Review and Gene Ontology Analysis | No Quality appraisal of primary studies |
| 9 | Kaul A | 2012 | Serum anti-glycan antibody biomarkers for inflammatory bowel disease diagnosis and progression: a systematic review and meta-analysis | No Quality appraisal of primary studies |
| 10 | Panés J, | 2011 | Systematic review: the use of ultrasonography, computed tomography and magnetic resonance imaging for the diagnosis, assessment of activity and abdominal complications of Crohn's disease | No Quality appraisal of primary studies |
| 11 | Kostakis ID | 2012 | Fecal calprotectin in pediatric inflammatory bowel disease: a systematic review | No Quality appraisal of primary studies |
| 12 | Greenup AJ | 2016 | Medical Imaging in Small Bowel Crohn's Disease-Computer Tomography Enterography, Magnetic Resonance Enterography, and Ultrasound: "Which One Is the Best for What?" | No Quality appraisal of primary studies |
| 13 | Menees SB | 2015 | A meta-analysis of the utility of C-reactive protein, erythrocyte sedimentation rate, fecal calprotectin, and fecal lactoferrin to exclude inflammatory bowel disease in adults with IBS | No diagnostic performance reported. |
| 14 | Barnes | 2016 | Are Anti-Tumor Necrosis Factor Trough Levels Predictive of Mucosal Healing in Patients With Inflammatory Bowel Disease?: A Systematic Review and Meta-Analysis | No diagnostic performance reported. |
| 15 | Šimurina | 2018 | Glycosylation of Immunoglobulin G Associates With Clinical Features of Inflammatory Bowel Diseases | No diagnostic performance reported. |
| 16 | Horsthuis K | 2008 | Inflammatory bowel disease diagnosed with US, MR, scintigraphy, and CT: meta-analysis of prospective studies | No diagnostic performance reported. |
| 17 | Horsthuis K | 2009 | Magnetic resonance imaging for evaluation of disease activity in Crohn's disease: a systematic review | No diagnostic performance reported. |
| 18 | Puylaert CA | 2015 | Grading of Crohn’s disease activity using CT, MRI, US and scintigraphy: a meta-analysis | No diagnostic performance reported. |
| 19 | Halligan S | 2016 | Prognostic biomarkers to identify patients destined to develop severe Crohn's disease who may benefit from early biological therapy: protocol for a systematic review, meta-analysis and external validation | No diagnostic performance reported. |
| 20 | Kopylov U | 2017 | Diagnostic yield of capsule endoscopy versus magnetic resonance enterography and small bowel contrast ultrasound in the evaluation of small bowel Crohn's disease: Systematic review and meta-analysis | No diagnostic performance reported. |
| 21 | Dong J | 2014 | Ultrasound as a diagnostic tool in detecting active Crohn's disease: a meta-analysis of prospective studies | No diagnostic performance reported. |
| 22 | Alrubaiy L | 2015 | Systematic Review of the Clinical Disease Severity Indices for Inflammatory Bowel Disease | Not a single non-invasive test |
| 23 | Goodsall TM | 2021 | Systematic Review: Gastrointestinal Ultrasound Scoring Indices for Inflammatory Bowel Disease | Not a single non-invasive test |
| 24 | Heida A | 2017 | Clinical Utility of Fecal Calprotectin Monitoring in Asymptomatic Patients with Inflammatory Bowel Disease: A Systematic Review and Practical Guide | Guideline statement |
| 25 | Brand EC | 2019 | Systematic Review and External Validation of Prediction Models Based on Symptoms and Biomarkers for Identifying Endoscopic Activity in Crohn's Disease | Not a single non-invasive test |
| 26 | Limsrivilai | 2017 | Meta-Analytic Bayesian Model For Differentiating Intestinal Tuberculosis from Crohn's Disease | Not a single non-invasive test |
| 27 | Lv | 2018 | Narrow Band Imaging for Surveillance in Inflammatory Bowel Disease: A Systematic Review and Meta-Analysis | Not a single non-invasive test |
| 28 | Freeman K | 2017 | Test accuracy of drug and antibody assays for predicting response to antitumour necrosis factor treatment in Crohn's disease: a systematic review and meta-analysis | Not our target condition |
| 29 | Stevens TW | 2018 | Systematic review: predictive biomarkers of therapeutic response in inflammatory bowel disease-personalised medicine in its infancy | Not our target condition |
| 30 | Nanda | 2012 | Impact of antibodies to infliximab on clinical outcomes and serum infliximab levels in patients with inflammatory bowel disease (IBD): a meta-analysis | Not our target condition |
| 31 | Vestito A | 2019 | Role of Ultrasound Elastography in the Detection of Fibrotic Bowel Strictures in Patients with Crohn's Disease: Systematic Review and Meta-Analysis | Not our target condition |
| 32 | Bettenworth D | 2019 | Assessment of Crohn's disease-associated small bowel strictures and fibrosis on cross-sectional imaging: a systematic review | Not our target condition |
| 33 | Xiong | 2014 | Serum antibodies to microbial antigens for Crohn's disease progression: a meta-analysis | Not our target condition |
| 34 | Church PC | 2014 | Systematic review with meta-analysis: magnetic resonance enterography signs for the detection of inflammation and intestinal damage in Crohn's disease | Not our target condition |
| 35 | Fauny | 2020 | Fecal calprotectin for the diagnosis of bowel inflammation in patients with rheumatological diseases: a systematic review | Not our target condition |
| 36 | Lee T | 2020 | Radiological outcomes in perianal fistulizing Crohn’s disease: A systematic review and meta-analysis | Not our target condition |
| 38 | Solon | 2013 | The effect of NOD2 polymorphism on postsurgical recurrence in Crohn's disease: a systematic review and meta-analysis of available literature | Genetic study |
| 39 | Mosli | 2013 | 10.1002/14651858.CD010848 | Protocol |
| 40 | Dulai | 2015 | MRI scoring indices for evaluation of disease activity and severity in Crohn's disease | Protocol |
| 41 | Yamada | 2019 | Systematic review and meta-analysis: risk of new onset IBD with the use of anti-interleukin-17 agents | Not our target condition |
| 42 | Simon EG | 2019 | Does fecal calprotectin equally and accurately measure disease activity in small bowel and large bowel Crohn's disease?: a systematic review | Not our target condition |
| 43 | Kedia | 2017 | Accuracy of computed tomographic features in differentiating intestinal tuberculosis from Crohn’s disease: a systematic review with meta-analysis | Not a single non-invasive test |
| 44 | Dulai | 2021 | Incorporating Fecal Calprotectin Into Clinical Practice for Patients With Moderate-to-Severely Active Ulcerative Colitis Treated With Biologics or Small-Molecule Inhibitors | Not our target condition |
| 45 | Xiang B | 2021 | The diagnostic and predictive value of fecal calprotectin and capsule endoscopy for small-bowel Crohn's disease: a systematic review and meta-analysis | Full text not found |
| 46 | Chavoshi M | 2021 | Diagnostic Accuracy of Magnetic Resonance Enterography in the Evaluation of Colonic Abnormalities in Crohn's Disease: A Systematic Review and Meta-Analysis | Full text not found |
| 47 | Li | 2016 | Fecal calprotectin for diagnosis of inflammatory bowel disease: A meta-analysis | Full text not found |
| 48 | Jin | 2017 | 10.4103/1319-3767.199135 | Full text not found |
| 49 | Xiang | 2021 | 10.1159/000514196 | Full text not found |
| 50 | Nguyen DL | 2015 | pANCA positivity predicts lower clinical response to infliximab therapy among patients with IBD | Full text not found |
| 51 | fraquelli | 2005 | Role of US in detection of Crohn disease: meta-analysis | No diagnostic performance reported. |
| 52 | Waugh N | 2013 | Faecal calprotectin testing for differentiating amongst inflammatory and non-inflammatory bowel diseases: systematic review and economic evaluation | Overlapping review Outdated |
| 53 | Van rheenen | 2010 | Faecal calprotectin for screening of patients with suspected inflammatory bowel disease: diagnostic meta-analysis | Overlapping review Outdated |
| 54 | Henderson | 2013 | The Diagnostic Accuracy of Fecal Calprotectin During  the Investigation of Suspected Pediatric Infl ammatory  Bowel Disease: A Systematic Review and Meta-Analysis | Overlapping review Outdated |
| 55 | Degraeuwe | 2015 | Faecal Calprotectin in Suspected Paediatric Inflammatory Bowel Disease | Overlapping review Outdated |
| 56 | Chen | 2013 | Effectiveness of interferon-gamma release assays for differentiating intestinal tuberculosis from Crohn’s disease: A meta-analysis | Overlapping review Outdated |
| 57 | Ma | 2015 | Contrast-enhanced ultrasound in the diagnosis of patients suspected of having active crohn’s disease: meta-analysis | Overlapping review Outdated |
| 58 | Wu | 2013 | Is Magnetic Resonance Imaging a Reliable Diagnostic Tool in the Evaluation of Active Crohn’s Disease in the Small Bowel? | Overlapping review Outdated |
| 59 | Kim | 2021 | Diagnostic Performance of Diffusion-weighted Imaging for Evaluation of Bowel Inflammation in Paediatric Inflammatory Bowel Disease: A Systematic Review and Meta-analysis | Full text not found |
| 60 | He | 2021 | Diagnostic performance of magnetic resonance enterography and ultrasound in children with inflammatory bowel diseases: a diagnostic test accuracy meta-analysis | Full text not found |
| 61 | Bromke | 2021 | Faecal Calprotectin in Assessment of Mucosal Healing in Adults with Inflammatory Bowel Disease: A Meta-analysis bowel diseases: a diagnostic test accuracy meta-analysis | No Quality appraisal of primary studies |
| 62 | Yan | 2020 | Aberrant expression of miR-21 in patients with inflammatory bowel disease A protocol for systematic review and meta analysis | No diagnostic performance reported |
| 63 | Mami, A. | 2020 | Faecal Calprotectin as Therapeutic Target in Patients with Crohn's Disease Treated with Anit-TNF: a Meta-analysis Of Individual Data | No Quality appraisal of primary studies |
| 64 | Tamilarasan | 2021 | Panenteric capsule endoscopy systems in ulcerative colitis: A systematic review and meta-analysis | Full text not found |
| 65 | Langley | 2021 | Inflammatory bowel disease and neutrophil–lymphocyte ratio: A systematic scoping review | No Quality appraisal of primary studies |
| 66 | Tamilarasan | 2022 | The diagnostic yield of pan-enteric capsule endoscopy in inflammatory bowel disease: A systematic review and meta-analysis | Abstract |

**Appendix 9:** AMSTAR 2 quality appraisal scores

| **Item No** | **1** | **2** | **3** | **4** | **5** | **6** | **7** | **8** | **9** | **10** | **11** | **12** | **13** | **14** | **15** | **16** | **Overall Rating** |
| --- | --- | --- | --- | --- | --- | --- | --- | --- | --- | --- | --- | --- | --- | --- | --- | --- | --- |
| Reese2006 | Y | N | N | Y | Y | Y | Y | Y | Y | N | Y | Y | Y | Y | N | N | Moderate |
| Von Roon2007 | Y | N | N | Y | N | Y | Y | Y | Y | N | Y | Y | Y | Y | Y | N | Moderate |
| Cui2008 | Y | N | N | Y | N | N | Y | PY | PY | N | N | N | Y | N | N | N | Low |
| Jellema2011 | Y | N | Y | N | Y | Y | Y | Y | Y | N | No MA | No MA | Y | Y | NO MA | N | Low |
| Mao2012 | Y | N | Y | Y | Y | Y | Y | Y | Y | N | Y | N | N | Y | Y | N | Low |
| Treglia2013 | Y | N | N | PY | Y | N | Y | Y | Y | N | Y | N | N | Y | Y | Y | Low |
| Giles2013 | Y | N | N | Y | N | N | Y | Y | Y | N | Y | N | Y | Y | N | N | Moderate |
| Qiu2014 | Y | N | N | Y | Y | Y | Y | Y | Y | N | Y | Y | Y | Y | Y | Y | Moderate |
| Zhou2014 | Y | N | N | Y | Y | Y | Y | Y | Y | N | Y | N | Y | Y | Y | Y | Moderate |
| Lin2014 | Y | N | N | Y | Y | N | Y | Y | Y | N | Y | N | N | Y | Y | Y | Low |
| Ng2014 | Y | N | N | Y | N | Y | Y | Y | Y | N | Y | Y | Y | Y | N | Y | Moderate |
| Zhang2014 | Y | N | Y | PY | N | Y | Y | Y | Y | N | Y | N | N | Y | Y | Y | Low |
| Qiu2015 | Y | N | Y | Y | Y | Y | Y | Y | Y | Y | N | Y | Y | Y | Y | Y | Moderate |
| Mosli2015 | Y | N | Y | Y | Y | Y | Y | Y | Y | N | Y | Y | Y | Y | Y | Y | Moderate |
| Wang2015 | Y | N | Y | Y | Y | Y | Y | Y | Y | N | Y | N | N | Y | N | Y | Low |
| Serafin2015 | Y | N | N | Y | Y | Y | Y | Y | Y | N | Y | N | N | N | Y | Y | Low |
| Holtman2015 | Y | N | N | Y | Y | Y | Y | Y | Y | N | Y | N | N | Y | N | Y | Low |
| Ahmed2016 | Y | N | N | Y | N | Y | Y | Y | Y | N | Y | Y | Y | Y | N | Y | Moderate |
| Choi2016 | Y | N | Y | Y | Y | Y | Y | Y | Y | N | Y | Y | N | Y | Y | N | Low |
| Xu2016 | Y | N | Y | Y | N | N | Y | PY | Y | N | Y | N | N | Y | Y | N | Low |
| Zhu2016 | Y | N | N | N | Y | Y | Y | Y | Y | N | Y | N | N | Y | Y | Y | Critially Low |
| Kopylov2016 | Y | N | Y | Y | N | Y | Y | Y | Y | N | Y | Y | Y | Y | N | Y | Moderate |
| Deng2017 | Y | N | N | Y | N | Y | N | Y | N | N | N | Y | Y | Y | Y | Y | Low |
| Holtman2017 | Y | N | N | Y | Y | Y | Y | Y | Y | N | Y | N | Y | Y | N | Y | Moderate |
| Yoon2017 | Y | N | N | Y | Y | Y | Y | Y | Y | N | Y | N | Y | Y | N | Y | Moderate |
| Yung2017 | Y | N | N | Y | N | Y | Y | Y | Y | N | Y | N | N | Y | N | Y | Low |
| Dai2018 | Y | N | N | Y | Y | Y | Y | Y | Y | Y | Y | N | N | Y | Y | Y | Low |
| Rispo2018 | Y | N | Y | Y | Y | Y | Y | Y | Y | N | Y | N | N | Y | Y | Y | Low |
| Tham2018 | Y | N | N | Y | N | Y | Y | Y | Y | N | Y | Y | Y | Y | N | Y | Moderate |
| Rokkas2018 | Y | N | N | Y | N | Y | N | Y | Y | N | Y | N | Y | Y | Y | Y | Moderate |
| Freeman2019 | Y | Y | Y | Y | Y | Y | Y | Y | Y | N | Y | N | N | Y | N | Y | Low |
| Li2019 | Y | N | Y | Y | Y | Y | Y | Y | Y | N | Y | Y | Y | Y | Y | Y | Moderate |
| Bellini2019 | Y | N | N | Y | Y | Y | Y | Y | Y | Y | Y | N | Y | Y | N | Y | Moderate |
| Petryszyn2019 | Y | N | N | Y | Y | Y | Y | Y | Y | N | Y | N | Y | Y | N | Y | Moderate |
| An2019 | Y | Y | N | Y | Y | N | Y | Y | Y | N | Y | N | N | N | N | Y | Low |
| van Wassenaer2019 | Y | N | N | PY | Y | N | Y | Y | Y | N | Y | N | Y | N | N | Y | Moderate |
| Gkiouras2020 | Y | Y | N | Y | Y | Y | Y | Y | Y | Y | Y | Y | N | Y | N | Y | Low |
| Lee2020 | Y | N | N | Y | Y | Y | Y | Y | Y | N | Y | N | Y | N | N | Y | Moderate |
| Dai2019 | Y | N | N | Y | Y | Y | Y | Y | Y | Y | Y | N | N | Y | Y | Y | Low |
| Sagami2020 | Y | Y | N | Y | Y | Y | Y | Y | Y | N | Y | Y | Y | Y | N | Y | Moderate |
| Jung2020 | Y | N | Y | PY | Y | N | Y | Y | Y | N | Y | Y | Y | Y | N | Y | Moderate |
| Treglia2017 | Y | N | N | Y | Y | N | Y | Y | Y | N | Y | N | N | Y | N | Y | Low |
| Bollegala2019 | Y | N | Y | Y | Y | N | PY | Y | Y | N | No MA | No MA | N | Y | No MA | N | Low |
| Ye2021 | Y | N | N | Y | Y | N | Y | Y | Y | N | Y | N | N | Y | N | Y | Low |
| Alshammari2021 | Y | Y | Y | PY | Y | Y | PY | PY | Y | Y | Y | Y | Y | Y | N | Y | Low |
| Sun2022 | Y | Y | N | PY | Y | Y | PY | PY | Y | Y | Y | Y | Y | Y | Y | Y | Moderate |
| Ahmad2021 | Y | PY | N | PY | N | Y | PY | PY | Y | Y | Y | Y | Y | Y | N | Y | Low |

No MA= No meta-analysis. PY=partial yes

­

**Appendix 10:** General characteristics of reviews with overlapping associations

| **Index** | **Study ID** | **AMSTAR 2 rating** | **Non-invasive test** | **Target condition** | **Population** | **Synthesis type (number)** | **CCA** | **Decision to retain ✓= Yes × = No** |
| --- | --- | --- | --- | --- | --- | --- | --- | --- |
| 1 | Holtman2015 | Moderate | FC | IBD vs non-IBD | Mixed | MA(10) | 0% slight | ✓ |
|  | Petryszyn 2019 | Moderate | FC | IBD vs non-IBD | Mixed | MA(19) |  | ✓ |
| 2 | Freeman2019 | Low | FC (cut-off 50) | IBD vs non-IBD | Mixed | MA(14) | 21.74% very high | × |
|  | Petryszyn 2019 | Moderate | FC (cut-off 50) | IBD vs non-IBD | Mixed | MA(15) |  | ✓ |
| 3 | Deng C2017 | Low | anti-GP2 | CD vs non-CD | Mixed | MA(10) | 36.6% very high | ✓ |
|  | Gkiouras2020 | Low | anti-GP2 | CD vs non-CD | Mixed | MA(5) |  | × |
| 4 | Deng C2017 | Low | anti-GP2-IgA | CD vs non-CD | Mixed | MA(9) | 27.78% very high | × |
|  | Gkiouras2020 | Low | anti-GP2-IgA | CD vs non-CD | Mixed | MA(14) |  | ✓ |
| 5 | Deng C2017 | Low | anti-GP2-IgG | CD vs non-CD | Mixed | MA(17) | 68.42% very high | ✓ |
|  | Gkiouras2020 | Low | anti-GP2-IgG | CD vs non-CD | Mixed | MA(15) |  | × |
| 6 | Rokkas2018 | Moderate | FC | Activity-UC | Mixed | MA(6) | 0% slight | ✓ |
|  | Ye2021 | Low | FC | Activity-UC | Mixed | review(4) |  | ✓ |
| 7 | Dai2018 | Low | FIT | Activity-UC | Mixed | MA(7) | 62.5% very high | × |
|  | Lee2020 | Moderate | FIT | Activity-UC | Mixed | MA(6) |  | ✓ |
| 8 | Zhu2016 | Critially Low | SICUS | Activity-SBCD | Mixed | MA(12) | 12.5% high | × |
|  | Kopylov2017 | Moderate | SICUS | Activity-SBCD | Mixed | MA(5) |  | ✓ |
| 9 | Kopylov2016 | Moderate | FC (cut-off 50) | Activity-SBCD | Mixed | MA(7) | 70% very high | × |
|  | Jung2020 | Moderate | FC (cut-off 50) | Activity-SBCD | Mixed | MA(10) |  | ✓ |
| 10 | Kopylov2016 | Moderate | FC (cut-off 100) | Activity-SBCD | Mixed | MA(5) | 41.67% very high | × |
|  | Jung2020 | Moderate | FC (cut-off 100) | Activity-SBCD | Mixed | MA(12) |  | ✓ |
| 11 | Kopylov2016 | Moderate | FC (cut-off 200) | Activity-SBCD | Mixed | MA(4) | 57.14% very high | × |
|  | Jung2020 | Moderate | FC (cut-off 200) | Activity-SBCD | Mixed | MA(7) |  | ✓ |
| 12 | Yung2017 | Low | US | Recurrence-postoperative CD | Mixed | MA(6) | 45.45% very high | × |
|  | Rispo2018 | Low | US | Recurrence-postoperative CD | Mixed | MA(10) |  | ✓ |
| 13 | Alshammari2021 | Low | US | Activity-IBD | Mixed | MA (4) | 4.55% slight | ✓ |
|  | Sagami2020 | Moderate | US | Activity-IBD | Mixed | MA (19) |  | ✓ |

MA = Meta-analysis, SBCD= small bowel Crohn's disease.

**Appendix 11:**

**A: List of studies included in analysis**

|  | **Author** | **Year** | **Title** |
| --- | --- | --- | --- |
| 1 | Reese GE | 2006 | Diagnostic precision of anti-Saccharomyces cerevisiae antibodies and perinuclear antineutrophil cytoplasmic antibodies in inflammatory bowel disease |
| 2 | von Roon AC | 2007 | Fecal calprotectin (FC) is a relatively new marker of intraluminal intestinal inflammation. Using meta-analytical techniques, the study aimed to evaluate the diagnostic precision of FC for inflammatory bowel disease (IBD) and colorectal cancer (CRC) in adults and children. |
| 3 | Cui | 2008 | Diagnostic value of antineutrophil cytoplasmic antibodies in patients with ulcerative colitis: a meta-analysis |
| 4 | Jellema P | 2011 | Inflammatory bowel disease: a systematic review on the value of diagnostic testing in primary care |
| 5 | Mao R | 2012 | Fecal calprotectin in predicting relapse of inflammatory bowel diseases: a meta-analysis of prospective studies |
| 6 | Treglia G | 2012 | Diagnostic performance of Fluorine-18-Fluorodeoxyglucose positron emission tomography in patients with chronic inflammatory bowel disease: a systematic review and a meta-analysis |
| 7 | Giles E | 2013 | Systematic review: MRI enterography for assessment of small bowel involvement in paediatric Crohn's disease |
| 8 | Qiu Y | 2014 | Systematic review with meta-analysis: magnetic resonance enterography vs. computed tomography enterography for evaluating disease activity in small bowel Crohn's disease |
| 9 | Zhou XL | 2014 | Fecal lactoferrin in discriminating inflammatory bowel disease from irritable bowel syndrome: a diagnostic meta-analysis |
| 10 | Lin JF | 2014 | Meta-analysis: fecal calprotectin for assessment of inflammatory bowel disease activity |
| 11 | Ng SC | 2014 | Systematic review with meta-analysis: accuracy of interferon-gamma releasing assay and anti-Saccharomyces cerevisiae antibody in differentiating intestinal tuberculosis from Crohn's disease in Asians |
| 12 | Zhang J | 2014 | Diagnostic performance of 18F-FDG-PET versus scintigraphy in patients with inflammatory bowel disease: a meta-analysis of prospective literature |
| 13 | Qiu Y | 2015 | Fecal calprotectin for evaluating postoperative recurrence of Crohn's disease: a meta-analysis of prospective studies |
| 14 | Mosli MH | 2015 | C-Reactive Protein, Fecal Calprotectin, and Stool Lactoferrin for Detection of Endoscopic Activity in Symptomatic Inflammatory Bowel Disease Patients: A Systematic Review and Meta-Analysis |
| 15 | Wang Y | 2015 | Diagnostic accuracy of fecal lactoferrin for inflammatory bowel disease: a meta-analysis |
| 16 | Holtman GA | 2015 | Noninvasive Tests for Inflammatory Bowel Disease: A Meta-analysis |
| 17 | Serafin Z | 2015 | Contrast-enhanced Ultrasound for Detection of Crohn's Disease Activity: Systematic Review and Meta-analysis |
| 18 | Ahmed O | 2016 | Magnetic Resonance Imaging of the Small Bowel in Crohn's Disease: A Systematic Review and Meta-Analysis |
| 19 | Choi SH | 2016 | Diffusion-weighted Magnetic Resonance Enterography for Evaluating Bowel Inflammation in Crohn's Disease: A Systematic Review and Meta-analysis |
| 20 | Xu H | 2016 | [A Meta-analysis of the accuracy of interferon-γ release assays in differentiating intestinal tuberculosis from Crohn's disease in Asia] |
| 21 | Zhu C | 2016 | Small intestine contrast ultrasonography for the detection and assessment of Crohn disease: A meta-analysis |
| 22 | Kopylov U | 2016 | Fecal calprotectin for the prediction of small-bowel Crohn's disease by capsule endoscopy: a systematic review and meta-analysis |
| 23 | Deng C | 2017 | Diagnostic value of the antiglycoprotein-2 antibody for Crohn's disease: a PRISMA-compliant systematic review and meta-analysis |
| 24 | Holtman GA | 2017 | Use of Laboratory Markers in Addition to Symptoms for Diagnosis of Inflammatory Bowel Disease in Children: A Meta-analysis of Individual Patient Data |
| 25 | Yoon HM | 2017 | Diagnostic Performance of Magnetic Resonance Enterography for Detection of Active Inflammation in Children and Adolescents With Inflammatory Bowel Disease: A Systematic Review and Diagnostic Meta-analysis |
| 26 | Yung DE | 2017 | Capsule Endoscopy, Magnetic Resonance Enterography, and Small Bowel Ultrasound for Evaluation of Postoperative Recurrence in Crohn's Disease: Systematic Review and Meta-Analysis |
| 27 | Rispo A | 2018 | Diagnostic Accuracy of Ultrasonography in the Detection of Postsurgical Recurrence in Crohn's Disease: A Systematic Review with Meta-analysis |
| 28 | Tham YS | 2018 | Fecal calprotectin for detection of postoperative endoscopic recurrence in Crohn's disease: systematic review and meta-analysis |
| 29 | Rokkas T | 2018 | Fecal calprotectin in assessing inflammatory bowel disease endoscopic activity: a diagnostic accuracy meta-analysis |
| 30 | Freeman K | 2019 | Faecal calprotectin to detect inflammatory bowel disease: a systematic review and exploratory meta-analysis of test accuracy |
| 31 | Li J | 2019 | Systematic Review with Meta-Analysis: Fecal Calprotectin as a Surrogate Marker for Predicting Relapse in Adults with Ulcerative Colitis |
| 32 | Bellini D | 2019 | Layered enhancement at magnetic resonance enterography in inflammatory bowel disease: A meta-analysis |
| 33 | Petryszyn P | 2019 | Faecal calprotectin as a diagnostic marker of inflammatory bowel disease in patients with gastrointestinal symptoms: meta-analysis |
| 34 | An YK | 2019 | Faecal calprotectin testing for identifying patients with organic gastrointestinal disease: systematic review and meta-analysis |
| 35 | van Wassenaer EA | 2019 | Diagnostic Accuracy of Transabdominal Ultrasound in Detecting Intestinal Inflammation in Paediatric IBD Patients-a Systematic Review |
| 36 | Gkiouras K | 2020 | Diagnostic and clinical significance of antigen-specific pancreatic antibodies in inflammatory bowel diseases: A meta-analysis |
| 37 | Lee MW | 2020 | Use of Fecal Occult Blood Testing as a Diagnostic Tool for Clinical Indications: A Systematic Review and Meta-Analysis |
| 38 | Dai C | 2020 | Fecal Lactoferrin for Assessment of Inflammatory Bowel Disease Activity: A Systematic Review and Meta-Analysis |
| 39 | Sagami S | 2020 | Accuracy of Ultrasound for Evaluation of Colorectal Segments in Patients With Inflammatory Bowel Diseases: A Systematic Review and Meta-analysis |
| 40 | Jung ES | 2020 | Diagnostic Accuracy of Fecal Calprotectin for the Detection of Small Bowel Crohn's Disease through Capsule Endoscopy: An Updated Meta-analysis and Systematic Review |
| 41 | Treglia | 2017 | Clinical role and accuracy of 18F-FDG PET in evaluating disease activity in patients with chronic inflammatory bowel disease: an updated systematic review and a bivariate meta-analysis |
| 42 | Bollegala | 2019 | Ultrasound vs Endoscopy, Surgery, or Pathology for the Diagnosis of Small Bowel Crohn's Disease and its Complications |
| 43 | Ye | 2021 | Can fecal calprotectin accurately identify histological activity of ulcerative colitis? A meta-analysis |
| 44 | Alshammari | 2021 | Diagnostic Accuracy of Non-Invasive Imaging for Detection of Colonic Inflammation in Patients with Inflammatory Bowel Disease: A Systematic Review and Meta-Analysis |
| 45 | Sun | 2022 | MicroRNAs as potential biomarkers for the diagnosis of inflammatory bowel disease: a systematic review and meta-analysis |
| 46 | Ahmad | 2021 | Magnetic resonance imaging in the management of Crohn's disease: a systematic review and meta-analysis |

**B: List of contemporary overlapping studies excluded from review**

|  | Author | Year | Title |
| --- | --- | --- | --- |
| 1 | Dai | 2018 | Fecal immunochemical test for predicting mucosal healing in ulcerative colitis patients: A systematic review and meta-analysis |

**Appendix 12:** Results of updating: meta-analysis

1. Results of updating Reese2006

| Diagnosis (IBD vs non-IBD): pANCA-IBD-Mixed | Se | Sp | AUC |
| --- | --- | --- | --- |
| Results from review | 0.33 (0.31–0.34) | 0.97 (0.96–0.98) | 0.87 |
| Results after update | 0.47 (0.28–0.66) | 0.85 (0.77–0.91) | 0.79 (0.76–0.83) |
| A change of more than 50% ? | No | No | No |

| Diagnosis (IBD vs non-IBD): ASCA IgA-IBD-Mixed | Se | Sp | AUC |
| --- | --- | --- | --- |
| Results from review | 0.31 (0.28–0.34) | 0.96 (0.94–0.97) | 0.821 |
| Results after update | 0.46 | 0.89 | / |
| A change of more than 50% ? | No | No | / |

| Diagnosis (IBD vs non-IBD): ASCA-IBD-Mixed | Se | Sp | AUC |
| --- | --- | --- | --- |
| Results from review | 0.40 (0.38–0.42) | 0.92 (0.91–0.94) | 0.78 |
| Results after update | 0.33 (0.15–0.58) | 0.92 (0.89–0.94) | 0.89 (0.86–0.92) |
| A change of more than 50% ? | No | No | No |

| Diagnosis (CD vs UC):  ASCA IgG-CD-Mixed | Se | Sp | AUC |
| --- | --- | --- | --- |
| Results from review | 0.46 (0.43–0.48) | 0.94 (0.92–0.95) | 0.85 |
| Results after update | 0.28 (0.14–0.48) | 0.92 (0.87–0.95) | 0.88 (0.85–0.91) |
| A change of more than 50% ? | No | No | No |

| Diagnosis (CD vs UC):  ASCA -CD-Mixed | Se | Sp | AUC |
| --- | --- | --- | --- |
| Results from review | 0.53 (0.51–0.56) | 0.89 (0.87–0.91) | 0.836 |
| Results after update | 0.45 (0.37–0.53) | 0.88 (0.84–0.92) | 0.77 (0.73–0.80) |
| A change of more than 50% ? | No | No | No |

| Diagnosis (CD vs UC):  pANCA -UC-Mixed | Se | Sp | AUC |
| --- | --- | --- | --- |
| Results from review | 0.55 (0.53–0.58) | 0.88 (0.87–0.90) | 0.818 |
| Results after update | 0.43 (0.36–0.51) | 0.83 (0.70–0.91) | 0.54 (0.50–0.59) |
| A change of more than 50% ? | No | No | No |

2. Results of Gile2013

| Diagnosis (IBD vs non-IBD): MRI-SBCD-Children | Se | Sp | AUC |
| --- | --- | --- | --- |
| Results from review | 0.82 (0.73–0.89) | 0.61 (0.51–0.71) | / |
| Results after update | 0.84 (0.77–0.89) | 0.63 (0.55–0.70) | 0.79 (0.75–0.83) |
| A change of more than 50% ? | No | No | / |

3. Results of Ahmed2016

| Activity:  MRI-SBCD-Children | Se | Sp | AUC |
| --- | --- | --- | --- |
| Results from review | 0.88 (0.86–0.91) | 0.88 (0.84–0.91) | / |
| Results after update | 0.92 (0.86–0.95) | 0.86 (0.78–0.92) | 0.95 (0.93–0.97) |
| A change of more than 50% ? | No | No | / |

4. Results of Kopylov2016

| Diagnosis (IBD vs non-IBD):  FC-SBCD-Mixed | Se | Sp | AUC |
| --- | --- | --- | --- |
| Results from review | 0.89 (0.68–0.97) | 0.55 (0.36–0.73) | / |
| Results after update | 0.87 (0.76–0.93) | 0.63 (0.49–0.76) | 0.84 (0.81–0.87) |
| A change of more than 50% ? | No | No | / |

5. Results of Yoon2017

| Activity:  MRI-IBD-Mixed | Se | Sp | AUC |
| --- | --- | --- | --- |
| Results from review | 0.83 (0.75–0.89) | 0.93 (0.90–0.95) | 0.95 (0.93–0.97) |
| Results after update | 0.84 (0.77–0.89) | 0.90 (0.82–0.95) | 0.93 (0.91–0.95) |
| A change of more than 50% ? | No | No | No |

| Activity:  MRI-IBD-Mixed-per patient | Se | Sp | AUC |
| --- | --- | --- | --- |
| Results from review | 0.86 (0.78–0.91) | 0.91 (0.82–0.96) | / |
| Results after update | 0.86 (0.77–0.91) | 0.92 (0.85–0.96) | / |
| A change of more than 50% ? | No | No | / |

| Activity:  MRI-IBD-Mixed-per segment | Se | Sp | AUC |
| --- | --- | --- | --- |
| Results from review | 0.72 (0.55–0.84) | 0.93 (0.90–0.95) | / |
| Results after update | 0.75 (0.59–0.87) | 0.88 (0.75–0.95) | / |
| A change of more than 50% ? | No | No | / |

6. Results of Rokkas2018

| Activity:  FC-IBD-Mixed | Se | Sp | AUC |
| --- | --- | --- | --- |
| Results from review | 0.85 (0.82–0.87) | 0.75 (0.71–0.79) | 0.88 |
| Results after update | 0.83 (0.80–0.86) | 0.77 (0.73–0.81) | 0.88 (0.84–0.90) |
| A change of more than 50% ? | No | No | No |

| Activity:  FC-UC-Mixed | Se | Sp | AUC |
| --- | --- | --- | --- |
| Results from review | 0.87 (0.85-0.89) | 0.77 (0.74-0.80) | 0.91 |
| Results after update | 0.86 (0.82–0.89) | 0.82 (0.77–0.85) | 0.91 (0.88–0.93) |
| A change of more than 50% ? | No | No | No |

Se=sensitivity, Sp=specificity

**Appendix 13:** Overview of updating

| **Systematic review** | **Search results** | **Screening results** | **Update or not** |
| --- | --- | --- | --- |
| Reese2006 | 506 | 15 | No※ |
| von Roon2007 | 1467 | 0 | No |
| Giles2013 | 258 | 1 | No |
| Qiu2014 | 82 | 0 | No |
| Zhou2014 | 286 | 0 | No |
| Nv2014 | 1490 | 0 | No |
| Qiu2015 | 537 | 5 | No |
| Mosli2015 | 125 | 0 | No |
| Ahmed2016 | 775 | 2 | No |
| Kopylov2016 | 24 | 3 | No |
| Yoon2017 | 114 | 2 | No |
| Tham2017 | 8 | 0 | No |
| Rokkas2018 | 87 | 1 | No |
| Li2019 | 18 | 0 | No |
| Bellini2019 | 68 | 0 | No |
| Petryszyn2019 | 204 | 0 | No |
| van Wassenaer2019 | 31 | 1 | No |
| Lee2020 | 288 | 0 | No |
| Jung2020 | 4 | 0 | No |

※: Since Reese et al did not specify which primary studies were included in each assessment, we could not obtain the data of each assessment to conduct a meta-analysis after update. We conducted a meta-analysis on the newly published studies without incorporating the original studies included in the Reese et al. Comparison of results with those presented in the review resulted in no statistically significant change or at least a 50% change in results. So a full update was not considered necessary.

**Table S1:** General characteristics of systematic reviews included in the umbrella review

| **Author/Year** | **Systematic review objective** | **Non-invasive test** | **Disease type** | **Target Condition** | **Population** | **Reference standard** | **Population source (number of participants)** | **Study designs (number of studies)** | **Quality Appraisal Tool** |
| --- | --- | --- | --- | --- | --- | --- | --- | --- | --- |
| Reese2006 | The aim of this study was to assess the diagnostic precision of anti-Saccharomyces cerevisiae (ASCA) and perinuclear antineutrophil cytoplasmic antibodies (pANCA) in inflammatory bowel disease (IBD) and evaluate their discriminative ability between ulcerative colitis (UC) and Crohn's disease (CD). | ASCA, pANCA | IBD | 1 | 3 | 1, 2, 3, 4 | 11608 | Prospective (44), retrospective (16) | STARD, QUADAS |
| Von Roon2007 | Fecal calprotectin (FC) is a relatively new marker of intraluminal intestinal inflammation. Using meta-analytical techniques, the study aimed to evaluate the diagnostic precision of FC for inflammatory bowel disease (IBD) and colorectal cancer (CRC) in adults and children. | FC | IBD | 1 | 1,2 | 1, 2, 3 | 5983 | NA (30) | QUADAS |
| Cui2008 | To evaluate the diagnostic value of antineutrophil cytoplasmic antibodies (ANCA) in patients with ulcerative colitis (UC) | ANCA | UC | 1 | 3 | NA | 381 | NA (10) | Self-specified |
| Jellema2011 | The clinical presentation of inflammatory bowel disease in primary care represents a diagnostic challenge as its symptoms are heterogeneous and common. To assist the primary care physician, we have summarized the available evidence on diagnostic tests in patients with abdominal symptoms. | CRP, ESR, ANCA-IgG, FC, FL, US | IBD | 1 | 3 | 1, 2, 3 | NA | cohort (13), case–control (10), both (1) | QUADAS |
| Mao2012 | To perform a meta-analysis of the predictive capacity of FC in IBD relapse. | FC | IBD | 3 | 3 | 1, 3 | 672 | Prospective (6) | QUADAS |
| Treglia2013 | To systematically review and meta-analyze published data about the diagnostic performance of Fluorine-18-Fluorodeoxyglucose ((18)F-FDG) positron emission tomography (PET) and PET/computed tomography (PET/CT) in patients with chronic inflammatory bowel disease (IBD). | CT | IBD | 1 | 3 | NA | 219 | NA (7) | The 2011 Oxford Center for Evidence-Based Medicine checklist for diagnostic studies |
| Giles2013 | To critically appraise the published evidence on MRE in the assessment of Paediatric inflammatory bowel disease by systematic review. | MRI | IBD | 1 | 2 | 1 | 496 | Prospective (7), retrospective (3), unclear (1) | QUADAS |
| Qiu2014 | To compare the overall diagnostic accuracy in assessing the activity of small bowel and complications. | MRI, CT | CD | 2 | 3 | 1, 5 | 290 | Prospective (6) | QUADAS-2 |
| Zhou2014 | To perform a meta-analysis evaluating the diagnostic ability of fecal lactoferrin (FL) to distinguish inflammatory bowel disease (IBD) from irritable bowel syndrome (IBS). | FL | IBD | 1 | 3 | 1, 2 | 1336 | NA (7) | QUADAS |
| Lin2014 | To evaluate the diagnostic accuracy of FC for differentiating between patients with active IBD and those in remission | FC | IBD | 2 | 3 | 1 | 1471 | Prospective (13) | QUADAS |
| Ng2014 | To assess the clinical usefulness of Interferon-gamma releasing assay (IGRA) and anti-Saccharomyces cerevisiae antibody (ASCA) in the diagnosis of ITB and CD, respectively. | IGRA, ASCA | IBD | 1 | 3 | 1, 2, 3, 4 | 1081 | cross-sectional (11) | QUADAS-2 |
| Zhang2014 | The aim of this study was to evaluate the diagnostic performance of fluorine-18 fluorodeoxyglucose-PET (F-FDG-PET), leukocyte scintigraphy (LS), and monoclonal antigranulocyte antibody scintigraphy (MAAS) in patients with inflammatory bowel disease (IBD) and perform pairwise comparisons of the diagnostic accuracy between these different imaging modalities. | 18F-FDG-PET, LS, MAAS | IBD | 2 | 1, 2, 3 | 1, 2 | NA | prospective (20) | QUADAS |
| Qiu2015 | To evaluate the utility of FC as a noninvasive marker of recurrence in patients with CD who had undergone previous surgical resection | FC | CD | 3 | 3 | 1, 2 | 613 | prospective (10) | QUADAS-2 |
| Mosli2015 | To reassess the diagnostic accuracy of FC in children with suspected IBD and developed an individual risk prediction rule using individual patient data. | FC | IBD | 2 | 3 | 1 | 2499 | Prospective (18), retrospective(1) | QUADAS-2 |
| Wang2015 | To do a systematic review using meta-analysis to assess the diagnostic accuracy of fecal lactoferrin (FL) in patients with inflammatory bowel disease (IBD). | FL | IBD | 1 | 3 | 1 | 1816 | prospective (5), retrospective (9) | QUADAS-2 |
| Serafin2015 | To verify the diagnostic value of CEUS in detecting aCD. | CEUS | CD | 2 | 3 | 1, 2, 3 | 332 | prospective (4), retrospective (1), unclear (3) | QUADAS-2 |
| Holtman2015 | To determine the diagnostic accuracy of symptoms, signs, noninvasive tests, and test combinations that can assist the clinician with the diagnosis of IBD in symptomatic children. | FC, CRP, ESR, PLT, Hb, Alb | IBD | 1 | 3 | 1, 2, 3 | 2806 | cohort (14), case-control (5) | QUADAS-2 |
| Ahmed2016 | We performed a systematic review and meta-analysis on the use of MR in detecting small bowel activity as well as extramural complications in Crohn's patients. | MRI | CD | 2 | 3 | 1, 2, 5 | 1020 | prospective (12), retrospective (2), both retrospective and prospective components (1), unclear (4) | QUADAS-2 |
| Choi2016 | To systematically determine the performance of diffusion-weighted imaging magnetic resonance enterography (DWI-MRE) for evaluating bowel inflammation in Crohn's disease and sources of heterogeneity between reported results. | DWI-MRI | CD | 2 | 3 | 1, 2, 4 | NA | Prospective (5), Retrospective (6) | QUADAS-2 |
| Xu2016 | This meta-analysis assessed the value of interferon-γ release assays (IGRAs) in the differential diagnosis of intestinal tuberculosis (ITB) from Crohn's disease (CD). | IGRA | CD | 1 | 3 | NA | 820 | NA (12) | QUADAS-2 |
| Zhu2016 | We aimed at evaluating the diagnostic accuracy of SICUS in the detection and assessment of small-bowel lesions and complications in CD | SICUS | SBCD | 2 | 3 | 2, 4, 5 | NA | NA (13) | QUADAS-2 |
| Kopylov2016 | To assess the diagnostic accuracy of calprotectin for the prediction of active small-bowel disease on capsule endoscopy by performing a diagnostic test meta-analysis. | FC | SBCD | 1, 2 | 3 | 1, 3 | 463 | prospective (3), retrospective (4) | QUADAS |
| Deng2017 | To perform a meta-analysis to evaluate the diagnostic performance of the antiglycoprotein-2 (GP2) antibody for Crohn's disease (CD). | anti-GP2 | CD | 1 | 3 | NA | 5623 | NA (17) | QUADAS |
| Holtman2017 | To perform a meta-analysis to evaluate the diagnostic performance of the antiglycoprotein-2 (GP2) antibody for Crohn's disease (CD). | CRP, ESR, PLT, Hb, Alb, FC | IBD | 1 | 2 | 2, 3 | 1120 | case-control (3), cohort (5) | QUADAS-2 |
| Yoon2017 | To evaluate the diagnostic performance of MR enterography for detection of active inflammation in children and adolescents with known or suspected IBD | MRI | IBD | 2 | 3 | 2 | 687 | prospective (9), retrospective (9) | QUADAS-2 |
| Yung2017 | To evaluate the accuracy of those modalities for detection of endoscopic recurrence in postoperative CD patients. | MRI, US | CD | 3 | 3 | 1 | 506 | NA (15) | QUADAS-2 |
| Rispo2018 | To assess the pooled diagnostic accuracy of US in the evaluation of PSR. | US | CD | 3 | 3 | 1 | 536 | prospective (10) | QUADAS-2 |
| Tham2018 | To evaluate the accuracy of common FC cut-offs for detection of endoscopic recurrence. | FC | CD | 3 | 3 | 1 | 588 | prospective (8), retrospective (1) | QUADAS-2 |
| Rokkas2018 | To determine the diagnostic performance of FC in assessing IBD endoscopic activity in adults. | FC | IBD | 2 | 3 | 1 | 3120 | prospective cohort (25) | QUADAS-2 |
| Freeman2019 | To assess the test accuracy of FC testing in primary care and compare it to secondary care estimates for the detection of inflammatory bowel disease (IBD). | FC | IBD | 1 | 3 | 1, 2, 4 | NA | Prospective (26), Retrospective (10), unclear (2) | QUADAS-2 |
| Li2019 | To detect the predictive value of FC for clinical relapse in adult UC patients based on updated literature | FC | UC | 3 | 3 | 1, 3 | 1110 | prospective (14) | QUADAS-2 |
| Bellini2019 | To systematically evaluate the accuracy of LP of contrast enhancement in the diagnosis of active inflammation in patients with CD. | MRI | CD | 2 | 3 | 1, 2, 5 | 245 | Prospective (2), retrospective (3) | QUADAS-2 |
| Petryszyn 2019 | To assess efficacy of faecal calprotectin as a diagnostic marker of IBD in patients with symptoms suggestive of such diagnosis | FC | IBD | 1 | 1, 3 | 1, 2 | 5032 | NA (19) | QUADAS |
| An2019 | To assess the clinical effectiveness of faecal calprotectin (FC) testing for distinguishing between organic gastrointestinal diseases (organic GID), such as inflammatory bowel disease (IBD), and functional gastrointestinal disorders (functional GIDs). | FC | IBD | 1 | 3 | 1, 4 | 6018 | prospective (16), retrospective (2) | QUADAS |
| van Wassenaer2019 | To assess the diagnostic test accuracy of transabdominal US in detecting intestinal inflammation in children with IBD in both diagnostic and follow-up settings | TAUS | IBD | 1, 2 | 2, 3 | 1, 4 | 424 | prospective (12), retrospective (2) | QUADAS-2 |
| Gkiouras2020 | To systematically review and meta-analyze evidence on the diagnostic accuracy of anti-GP2 tests in patients with suspected/confirmed CD. | anti-GP2 | CD | 1 | 3 | 1, 2, 3, 4 | NA | NA (15) | QUADAS-2 |
| Lee2020 | To assess performance characteristics of FOBT as a diagnostic test for clinical indications. | FIT | UC | 2 | 3 | 1 | NA | Prospective (13), Retrospective (9) | QUADAS-2 |
| Dai2019 | To perform a meta-analysis evaluating the diagnostic accuracy of fecal lactoferrin (FL) in assessing IBD activity | FL | IBD | 2 | 3 | 1, 3 | NA | prospective (10) | QUADAS-2 |
| Sagami2020 | We evaluated the diagnostic accuracy of ultrasound in different colorectal segments of patients with IBD. | US | IBD | 2 | 1, 2, 3 | 1 | 504 | NA (7) | QUADAS-2 |
| Jung2020 | To evaluate the diagnostic accuracy of FC to detect active small bowel inflammation observed during capsule endoscopy. | FC | SBCD | 1, 2 | 3 | 1, 3 | NA | prospective (8), retrospective (6) | QUADAS-2 |
| Treglia2017 | To perform an updated systematic review on the clinical role of 18F FDG PET in evaluating disease activity in patients with IBD and a bivariate meta-analysis on its accuracy in this setting. | PET/CT | IBD | 2 | 3 | NA | 313 | Prospective (5), Retrospective (1), unclear (1) | The 2011 Oxford Center for Evidence-Based Medicine checklist for diagnostic studies |
| Bollegala2019 | To determine the accuracy of ultrasound in diagnosing SBCD and its complications as compared with endoscopic visualization, surgery, and/or pathology. | US | SBCD | 2, 3 | 3 | 1, 2, 5 | NA | prospective cohort (22) | QUADAS |
| Ye2021 | To determine the accuracy of FC for evaluating histological activity of UC, based on updated definitions | FC | UC | 2 | 1 | 2 | 1491 | prospective (8), Post hoc (1) | QUADAS |
| Alshammari2021 | Non-invasive colonic imaging using bowel ultrasound (US), computed tomography (CT), and magnetic resonance imaging (MRI) may have a role in quantifying colonic disease activity. We reviewed the diagnostic accuracy of these modalities for assessment of endoscopically or histopathologically defined colonic disease activity in IBD. | US, MRI | IBD | 2 | 3 | 1 | 2836 | 18 | QUADAS-2 |
| Sun2022 | The clinical importance of aberrantly expressed microRNAs (miRNAs) in diagnosing inflammatory bowel disease (IBD) has not been well established, so was investigated in this systematic review and meta-analysis. | microRNAs | IBD | 1 | 3 | 2 | 1644 | 15 | QUADAS-2 |
| Ahmad2021 | This study reports on a systematic review and meta-analysis of magnetic resonance enterography for the prediction of CD activity and evaluation of outcomes and possible complications. | MRI | CD | 2 | 3 | 4 | 1470 | 19 | QUADAS-II |

Target Condition: 1=diagnosis, 2=activity, 3=recurrence

Reference standard: 1=endoscopic, 2=histological, 3=clinical, 4=radiological, 5=surgery

Population: 1=adults, 2=children, 3=mixed

NA=not available,

**Table S2:** Tabular presentation of findings: Meta-analysis

| **Target condition (disease type + population)** | **Author/Year** | **Non-invasive test** | **No of studies included** | **Participants** | **Diagnostic performance** | | | | | | **Overall evidence of publication bias** | **AMSTAR2 rating** |
| --- | --- | --- | --- | --- | --- | --- | --- | --- | --- | --- | --- | --- |
|  |  |  |  |  | **Se** | **I²-Se** | **Sp** | **I²-Sp** | **AUC** | **SE-AUC** |  |  |
| **Diagnosis-IBD vs non-IBD** | | | | | | | | | | | | |
| IBD-mixed | Holtman2015 | FC | 10 | 867 | 0.99 (0.92–1.00) | NA | 0.65 (0.54–0.74) | NA | NA | NA | NA | Low |
|  |  | CRP | 9 | 1146 | 0.63 (0.51–0.73) | NA | 0.88 (0.80–0.93) | NA | NA | NA |  |  |
|  |  | ESR | 11 | 1434 | 0.66 (0.58–0.73) | NA | 0.84 (0.80–0.88) | NA | NA | NA |  |  |
|  |  | PLT | 8 | 732 | 0.55 (0.36–0.73) | NA | 0.88 (0.81–0.93) | NA | NA | NA |  |  |
|  |  | Hb | 9 | 1454 | 0.37 (0.24–0.52) | NA | 0.90 (0.83–0.94) | NA | NA | NA |  |  |
|  |  | Alb | 6 | 527 | 0.48 (0.31–0.66) | NA |  | NA | NA | NA |  |  |
|  | Petryszyn2019 | FC | 19 | 5032 | 0.882 (0.827–0.921) | NA | 0.799 (0.693–0.875) | NA | NA | NA | NA | Moderate |
|  |  | FC-cutoff 50 | 14 | NA | 0.850 (0.605–0.955) | NA | 0.847 (0.647–0.943) | NA | NA | NA |  |  |
|  | Freeman2019 | FC-cutoff 100 | 5 | NA | 0.72 (0.63–0.80) | NA | 0.82 (0.78-0.86) | NA | NA | NA | NA | Low |
|  | Reese2006 | ASCA-IgA | 3 | 1651 | 0.314 (0.285–0.345) | NA | 0.96 (0.943–0.973) | NA | 0.821 | 0.036 | NA | Moderate |
|  |  | ASCA | 13 | 4097 | 0.397 (0.376–0.418) | NA | 0.925 (0.913–0.937) | NA | 0.783 | 0.026 |  |  |
|  |  | pANCA | 27 | 6117 | 0.328 (0.312–0.344) | NA | 0.971 (0.964–0.977) | NA | 0.872 | 0.012 |  |  |
|  | Wang2015 | FL | 14 | 1092 | 0.82 (0.72–0.89) | NA | 0.95 (0.88–0.98) | NA | 0.95 (0.93–0.97) | NA | NA | Low |
|  | Jellema2011 | US | 4 | NA | 0.73 (0.65–0.80) | NA | 0.95 (0.91–0.97) | NA | NA | NA | NA | Low |
|  | Treglia2012 | PET/CT  (per segment) | 7 | NA | 0.85 (0.81–0.88) | 79.7% | 0.87 (0.84–0.90) | 91.6% | 0.933 | 0.0314 | NA | Low |
|  | Sun2021 | miRNA | 15 | 1644 | 0.80 (0.79–0.82) | 86.0% | 0.84 (0.82–0.86) | 79.9% | 0.89 | NA | <0.10 | Moderate |
| IBD-adults | Petryszyn2019 | FC | 5 | NA | 0.825 (0.661–0.920) | NA | 0.900 (0.573–0.984) | NA | NA | NA | NA | Moderate |
| IBD-children | Holtman2017 | FC | 6 | NA | NA | NA | NA | NA | 0.95 (0.93–0.98) | NA | NA | Moderate |
|  |  | CRP | 8 | NA | NA | NA | NA | NA | 0.79 (0.73–0.85) | NA |  |  |
|  |  | ESR | 8 | NA | NA | NA | NA | NA | 0.84 (0.82–0.87) | NA |  |  |
|  |  | PLT | 6 | NA | NA | NA | NA | NA | 0.79 (0.75–0.83) | NA |  |  |
|  |  | Hb | 5 | NA | NA | NA | NA | NA | 0.76 (0.71–0.80) | NA |  |  |
|  |  | Alb | 5 | NA | NA | NA | NA | NA | 0.82 (0.73–0.90) | NA |  |  |
|  | von Roon2007 | FC-cutoff 50 | 3 | 201 | 0.83 (0.73 –0.90) | NA | 0.85 (0.77 –0.91) | NA | 0.96 | 0.02 | NA | Moderate |
| UC-mixed | von Roon2007 | FC | 2 | 235 | 0.78 (0.69–0.86) | NA | 0.78 (0.70 –0.84) | NA | NA | NA | NA | Moderate |
|  | Cui2008 | ANCA | 10 | 381 | 0.522 | NA | 0.99 | NA | NA | NA | NA | Low |
|  | Wang2015 | FL | 5 | 511 | 0.82 (0.67–0.91) | NA | 1.00 (0.67–1.00) | NA | 0.94 (0.91–0.96) | NA | NA | Low |
| CD-children | von Roon2007 | FC-cutoff 50 | 2 | 119 | 0.97 (0.86–1.00) | NA | 0.79 (0.69 –0.87) | NA | NA | NA | NA | Moderate |
|  |  | FC-cutoff 100 | 2 | 155 | 1.00 (0.93–1.00) | NA | 0.98 (0.93 –1.00) | NA | NA | NA |  |  |
|  |  | FC-cutoff 150 | 5 | 733 | 0.95 (0.92–0.97) | NA | 0.84 (0.80 –0.87) | NA | NA | NA |  |  |
| CD-mixed | Wang2015 | FL | 4 | 534 | 0.75 (0.65–0.84) | NA | 1.00 (0.50–1.00) | NA | 0.84 (0.81–0.87) | NA | NA | Low |
|  | Deng2017 | anti-GP2 (either IgG or IgA) | 10 | 4013 | 0.24 (0.18–0.32) | 90% | 0.96 (0.93–0.97) | 82% | 0.72 (0.68–0.76) | NA | 0.22 | Low |
|  |  | anti-GP2 IgA | 14 | 3914 | 0.15 (0.12–0.18) | NA | 0.97 (median) | NA | NA | NA | NA |  |
|  |  | anti-GP2 IgG | 17 | 5630 | 0.19 (0.14–0.25) | 91% | 0.97 (0.94–0.98) | 78% | 0.71 (0.67-0.75) | NA | 0.11 |  |
| SBCD-mixed | Kopylov2016 | FC | 5 | 305 | 0.89 (0.68–0.97) | NA | 0.55 (0.36–0.73) | NA | NA | NA | NA | Moderate |
| SBCD-children | Giles2013 | MRI | 6 | NA | 0.84 (0.77–0.90) | NA | 0.97 (0.91–0.99) | NA | 0.95 | NA | NA | Moderate |
| **Diagnosis-IBD vs IBS** | | | | | | | | | | | | |
| IBD-mixed | Freeman2019 | FC-cutoff 50 | 11 | NA | 0.97 (0.91–0.99) | NA | 0.76 (0.66–0.84) | NA | NA | NA | NA | Low |
|  |  | FC-cutoff 100 | 5 | NA | 0.92 (0.85–0.96) | NA | 0.86 (0.82–0.89) | NA | NA | NA | NA |  |
|  | Zhou2014 | FL | 7 | NA | 0.78 (0.75–0.82) | 49.7% | 0.94 (0.91–0.96) | 67.9% | 0.94 | 0.02 | <0.05 | Moderate |
| **Diagnosis-IBD vs FGID** | | | | | | | | | | | | |
| IBD-adults | An2019 | FC | 10 | 2719 | 0.88 (0.80–0.93) | NA | 0.72 (0.59–0.82) | NA | 0.89 | NA | NA | Low |
| **Diagnosis-CD vs ITB** | | | | | | | | | | | | |
| CD-mixed | Xu2016 | IGRAs | 12 | 820 | 0.828 (0.784–0.855) | 72.2% | 0.867 (0.832–0.896) | 41.4% | NA | NA | 11.18 | Low |
|  | Ng2014 | ASCA | 6 | NA | 0.33 (0.27–0.38) | NA | 0.83 (0.77–0.88) | NA | 0.58 | NA | NA | Moderate |
| **Diagnosis-CD vs UC** | | | | | | | | | | | | |
| UC-mixed | Reese2006 | pANCA | 31 | 4054 | 0.553 (0.530–0.576) | NA | 0.885 (0.871–0.898) | NA | 0.818 | 0.017 | NA | Moderate |
| CD-mixed | Gkiouras2020 | anti-GP2 (IgA and/or IgG) | 5 | 1541 | 0.20 (0.04–0.35) | NA | 0.97 (median) | NA | NA | NA | NA | Low |
|  |  | anti-GP2 IgG | 15 | 3947 | 0.30 (0.24–0.36) | NA | 0.93 (median) | NA | NA | NA |  |  |
|  |  | anti-GP2 IgA | 14 | 3497 | 0.11 (0.03–0.20) | NA | 0.98 (median) | NA | NA | NA |  |  |
|  | Reese2006 | ASCA | 16 | 2761 | 0.533 (0.508–0.557) | NA | 0.892 (0.872–0.910) | NA | 0.836 | 0.018 | NA | Moderate |
|  |  | ASCA IgG | 16 | 2492 | 0.457 (0.432–0.483) | NA | 0.935 (0.917–0.949) | NA | 0.85 | 0.013 |  |  |
|  |  | ASCA IgA | 13 | 2128 | 0.408 (0.381–0.435) | NA | 0.955 (0.938–0.967) | NA | 0.863 | 0.077 |  |  |
| **Activity** | | | | | | | | | | | | |
| IBD-mixed | Treglia2017 | PET/CT (per segment) | 9 | 313 | 0.856 (0.76–0.92) | NA | 0.855 (0.75–0.92) | NA | NA | NA | NA | Low |
|  | Sagami2020 | US (per bowel) | NA | NA | 0.864 (0.761–0.927) | NA | 0.883 (0.581–0.976) | NA | NA | NA | NA | Moderate |
|  | Yoon2017 | MRI | 18 | 687 | 0.83 (0.75–0.89) | 84.1% | 0.93 (0.90–0.95) | 68.8% | 0.95 (0.93–0.97) | 0.13 | NA | Moderate |
|  |  | MRI-per patient | 13 | NA | 0.86 (0.78–0.91) | 54.9% | 0.91 (0.82–0.96) | 56.9% | NA | NA |  |  |
|  |  | MRI-per lesion | 7 | NA | 0.72 (0.55–0.84) | 91.9% | 0.93 (0.90–0.95) | 62.9% | NA | NA |  |  |
|  | Zhang2014 | LS (per patient) | 7 | 329 | 0.91 (0.87–0.95) | 61.5% | 0.85 (0.76–0.91) | 43.8% | NA | NA | 0.936 | Low |
|  |  | LS (per bowel) | 9 | 998 | 0.79 (0.76–0.82) | 27.2% | 0.86 (0.82–0.89) | 62.8% | NA | NA | 0.007 |  |
|  | Rokkas2018 | FC | 25 | 3120 | 0.85 (0.82–0.87) | 69.87% | 0.75 (0.71–0.79) | 73.41% | 0.88 (0.85–0.90) | NA | 0.29 | Moderate |
|  | Lin2014 | FC-cutoff 50 | 5 | 693 | 0.92 (0.90–0.94) | 65.9% | 0.60 (0.52–0.67) | 90.5% | NA | NA | 0.425 | Low |
|  |  | FC-cutoff 100 | 5 | 559 | 0.84 (0.80–0.88) | 50.7% | 0.66 (0.59–0.73) | 83.5% | NA | NA |  |  |
|  |  | FC-cutoff 250 | 7 | 763 | 0.80 (0.76–0.84) | 81.3% | 0.82 (0.77–0.86) | 58.0% | NA | NA |  |  |
|  | Mosli2015 | CRP | 4 | NA | 0.49 (0.34–0.64) | NA | 0.92 (0.72–0.98) | NA | 0.72 (0.68–0.76) | NA | NA | Moderate |
|  | Alshammari2021 | US | 4 | 660 | 0.82 | 86.70% | 0.9 | 0.00% | 0.90 (0.75 - 1.00) | NA | NA | Low |
|  |  | MRI | 9 | 2176 | 0.75 | 86.50% | 0.91 | 88.40% | 0.88 (0.82 - 0.93) | NA | NA |  |
| IBD-adults | Zhang2014 | PET/CT (per bowel) | 3 | NA | 0.84 (0.78–0.90) | 68.0% | 0.86 (0.81–0.90) | 94.0% | NA | NA | 0.555 | Low |
|  |  | MAAS | 3 | 328 | 0.45 (0.37–0.53) | 70.4% | 0.94 (0.89–0.97) | 84.7% | NA | NA | 0.127 |  |
|  | Sagami2020 | US (per bowel) | NA | 1341 | 0.86 (0.745–0.928) | NA | 0.836 (0.533–0.958) | NA | NA | NA | NA | Moderate |
| IBD-children | Sagami2020 | US (per bowel) | NA | 150 | 0.876 (0.542–0.977) | NA | 1 | NA | NA | NA | NA | Moderate |
| UC-mixed | Sagami2020 | US (per bowel) | NA | 1222 | 0.886 (0.800–0.939) | NA | 0.819 (0.456–0.961) | NA | NA | NA | NA | Moderate |
|  | Rokkas2018 | FC | NA | NA | 0.873 (0.854–0.891) | NA | 0.771 (0.737–0.803) | NA | 0.91 | NA | NA | Moderate |
|  | Ye2021 | FC | 4 | 591 | 0.76 (0.71–0.81) | 0% | 0.71 (0.62–0.78) | 44% | 0.79 (0.75–0.82) | NA | NA | Low |
|  | Dai2020 | FL | 6 | 356 | 0.81 (0.64-0.92) | NA | 0.82 (0.61-0.93) | NA | 0.88 (0.85-0.91) | NA | 0.355 | Low |
|  | Lee2020 | FIT | 6 | NA | 0.72 (0.57–0.84) | NA | 0.8 (0.67–0.89) | NA | NA | NA | NA | Moderate |
| CD-mixed | Sagami2020 | US (per bowel) | NA | 269 | 0.725 (0.454–0.894) | NA | 0.977 (0.700–0.999) | NA | NA | NA | NA | Moderate |
|  | Serafin2015 | CEUS | 8 | 332 | 0.94 (0.87–0.97) | NA | 0.79 (0.67–0.88) | NA | 0.94 | NA | NA | Low |
|  | Ahmed2016 | MRI | 19 | 1020 | 0.88 (0.86–0.91) | 77.6% | 0.88 (0.84–0.91) | 67.3% | 0.93 | NA | NA | Moderate |
|  | Choi2016 | DWI-MRE | 9 | NA | 0.929 (0.858–0.966) | 87.9% | 0.91 (0.797–0.963) | 95.1% | NA | NA | 0.14 | Low |
|  | Bellini2019 | MRI-LP | 5 | 245 | 0.493 (0.410–0.578) | 90.7% | 0.891 (0.813–0.944） | 48.6% | 0.82 | 0.06 | NA | Moderate |
|  | Rokkas2018 | FC | NA | NA | 0.824 (0.802–0.844) | NA | 0.721 (0.69–0.75) | NA | 0.84 | NA | NA | Moderate |
|  | Dai2020 | FL | 6 | 236 | 0.82 (0.73–0.88) | NA | 0.71 (0.63–0.78) | NA | 0.84 (0.80–0.87) | NA | 0.686 | Low |
| SBCD-mixed | Qiu2014 | CT | 6 | NA | 0.86 (0.79–0.91) | 61.8% | 0.84 (0.75–0.90) | 67.0% | 0.91 | 0.03 | NA | Moderate |
|  | Ahmad2021 | MRI | 19 | 1470 | 0.9 | 80.30% | 0.89 | 70.00% | NA | NA | NA | Low |
|  | Jung2020 | FC-cutoff 50 | 10 | 794 | 0.831 (0.740–0.895) | NA | 0.502 (0.359–0.644) | NA | 0.774 | NA | NA | Moderate |
|  |  | FC-cutoff 100 | 12 | 961 | 0.725 (0.657–0.784) | NA | 0.728 (0.622–0.814) | NA | 0.763 | NA |  |  |
|  |  | FC-cutoff 200 | 7 | 594 | 0.495 (0.361–0.629) | NA | 0.882 (0.738–0.952) | NA | 0.67 | NA |  |  |
| **Recurrence** | | | | | | | | | | | | |
| IBD-mixed | Mao2012 | FC | 6 | 672 | 0.78 (0.72–0.83) | NA | 0.73 (0.68–0.77) | NA | 0.83 | NA | <0.05 | Low |
| UC-mixed | Li2019 | FC | 10 | 1110 | 0.75 (0.70–0.79) | NA | 0.77 (0.74–0.80) | NA | 0.82 | 0.027 | NA | Moderate |
| CD-mixed | Mao2012 | FC | 6 | 354 | 0.75 (0.64–0.84) | NA | 0.71 (0.64–0.76) | NA | 0.79 | 0.05 | <0.05 | Low |
|  | Qiu2015 | FC-postoperative-ER | 8 | 391 | 0.82 (0.73–0.89) | 51.9% | 0.61 (0.51–0.71) | 88.8% | 0.77 (0.74–0.81) | NA | 0.79 | Moderate |
|  |  | FC-postoperative-CR | 3 | 183 | 0.59 (0.47–0.71) | NA | 0.88 (0.80–0.93) | NA | 0.97 | 0.04 |  |  |
|  | Tham2018 | FC- postoperative- cutoff 50 | 7 | 528 | 0.90 (0.83–0.96) | 73% | 0.36 (0.25–0.47) | 66.6% | 0.72 | NA | NA | Moderate |
|  |  | FC- postoperative- cutoff 100 | 9 | 588 | 0.81 (0.71–0.91) | 83.1% | 0.57 (0.48–0.64) | 38.2% | 0.67 | NA |  |  |
|  |  | FC- postoperative- cutoff 150 | 6 | 340 | 0.70 (0.59–0.81) | 51.8% | 0.69 (0.61–0.77) | 24.9% | 0.73 | NA |  |  |
|  |  | FC- postoperative- cutoff 200 | 6 | 284 | 0.55 (0.43–0.69) | 49.7% | 0.71 (0.62–0.79) | 48.7% | 0.69 | NA |  |  |
|  | Rispo2018 | US | 10 | 536 | 0.94 (0.86–0.97) | 48% | 0.84 (0.62–0.94) | 51% | 0.9 | NA | NA | Low |
|  |  | BS | 5 | 219 | 0.82 (0.76–0.88) | 0% | 0.88 (0.74–0.95) | 38% | 0.875 |  |  |  |
|  |  | SICUS | 6 | 257 | 0.99 (0.99–1.00) | 39% | 0.74 (0.73–0.74) | 66% | 0.92 |  |  |  |
|  | Yung2017 | MRI | 3 | 76 | 0.973 (0.891–0.998) | 0% | 0.837 (0.616–0.959) | 18.4% | 0.9767 | 0.0310 | NA | Low |
| SBCD-mixed | Zhu2016 | SICUS- postoperative | 2 | NA | 0.899 (0.817–0.953) | NA | 0.808 (0.606–0.934） | NA | NA | NA | NA | Critially Low |

NA=not available, Se=sensitivity, Sp=specificity.

**Table S3:** Tabular presentation of findings: Narrative syntheses

| **Target condition (disease type + population)** | **Study identity**  **Author/Year** | **Non-invasive test** | **Participants** | **Narrative summary**  **Diagnostic performance (95%CI)** | **Reviewer’s conclusions** | **Overall AMSTAR 2 rating for Review** |
| --- | --- | --- | --- | --- | --- | --- |
| **Diagnosis-IBD vs non-IBD** | | | | | | |
| UC-adults | Jellema2011 | ANCA IgG | 40 | Duerr1991 [32]:  (UC+ vs functional diarrhoea)  Se 0.67 (0.54–0.79), Sp 0.85 (0.70–0.94) | Not reported | Low |
| IBD-children | van Wassenaer2019 | TAUS-per patient | 66 | Alberini2001 (16)：  Se 0.39 (0.20–0.61), Sp=0.90 (0.68–0.99);  de Ridder 2012 (20)：  Se 0.54 (0.25–0.81), Sp1.00（0.54–1.00）；  Ziech2014 (22)：  Se 0.55, Sp 1.00. | This systematic review shows that the diagnostic accuracy of US in detecting intestinal inflammation as seen on MRE and/or ileo colonoscopy in paediatric IBD patients remains inconclusive. | Moderate |
| **Diagnosis-IBD vs IBS** | | | | | | |
| IBD-mixed | Jellema2011 | S100A12 | 83 | Kaiser2007 [34]:  (S100A12 > 0.8 mg/kg, Active IBD+ vs IBS+)  Se 0.86 (0.73–0.94), Sp 0.96 (0.79–0.99) | Not reported | Low |
| **Activity** | | | | | | |
| IBD-children | van Wassenaer2019 | TAUS-per patient | 162 | 18-Bremner2006:  Se 0.48, Sp 0.93;  19-Civitelli2014:  Se 1, Sp 0.93;  23-Faure1997:  Se 0.88, Sp 0.93;  21-Haber2002:  Se 0.77, Sp 0.83 | This systematic review shows that the diagnostic accuracy of US in detecting intestinal inflammation as seen on MRE and/or ileo colonoscopy in paediatric IBD patients remains inconclusive. | Moderate |
|  |  | TAUS-per bowel | 177 | 17-Borthne 2006:  Term. Ileum: Se 0.93(0.70–0.99)  18-Bremner 2006:  Cecum: Se 0.33, Sp 1.00;  Asc. Colon: Se 0.46, Sp 0.88;  Tran. Colon: Se 0.67, Sp 0.90;  Desc. Colon: Se 0.54, Sp 1.00;  Sigmoid：Se 0.50, Sp 0.88;  19-Civitelli 2014:  Asc. Colon: Se 0.75 (0.42–0.93), Sp 1 (0.74–1);  Tran. Colon: Se 0.86 (0.60–0.97), Sp 1 (0.70-1.00)；Desc. Colon: Se 0.96 (0.80-1.00), Sp 1 (0.62-0.97)；  23-Faure 1997:  Term. Ileum: Se 1, Sp 0.92;  Asc. Colon: Se 0.88, Sp 0.92;  Tran. Colon: Se 0.80, Sp 0.90;  Desc. Colon: Se 0.93, Sp 1.00;  Rectum: Se 0.89  21-Haber 2002:  Ter. Ileum: Se 1.00, Sp 0.72;  Asc. Colon: Se 0.72, Sp 0.81;  Tran. Colon: Se 0.74, Sp 0.94;  Desc. Colon: Se 0.74, Sp 0.89 |  |  |
|  | Zhang2014 | PET/CT-per patient | 65 | Lemberg2005 [31]:  SBFT was used as the reference standard:  Se 0.59 (0.36–0.79), Sp 1.00 (0.77–1.00)  colonoscopy was used as the reference standard:  Se 0.86 (0.70–0.95), Sp 0.50 (0.01–0.99) | NA | Low |
| IBD-mixed | van Wassenaer2019 | TAUS-per patient | 111 | 24-Ahmad 2016:  Se 0.64 (0.45–0.80);  27-Barber 2017:  Se 0.81 (0.70–0.89), Sp 0.95(0.92–0.97);  29-Dagia 2008:  Se 0.71 (0.29–0.96), Sp 1.00 (0.16–1.00);  25-Magnano 2003:  Se 0.93 (0.68–1.00), Sp 1.00 (0.48-1.00) | This systematic review shows that the diagnostic accuracy of US in detecting intestinal inflammation as seen on MRE and/or ileo colonoscopy in paediatric IBD patients remains inconclusive. | Moderate |
|  |  | TAUS-per bowel | 70 | 26-Dillman 2016:  Distal small Bowel: Se 0.83, Sp 0.71;  28-Tsai 2017：  Term. Ileum: Se 0.67, Sp 0.78 (rater1);  Se 0.83, Sp 0.78 (rater2) |  |  |
| SBCD-mixed | Bollegala2019 | US | NA | TAUS:  Se 0.54–0.93, Sp 0.97–1.00;  CEUS (when compared with ileocolonoscopy):  Se 0.94–1.00, Sp 0.79–0.92 | Overall, based on these studies, it remains unclear whether contrast enhancement (CE) and/or Doppler reliably enhance diagnostic accuracy.Overall, TAUS, compared with ileocolo noscopy, yielded the most optimal operating character | Low |
| **Recurrence** | | | | | | |
| SBCD-mixed | Bollegala2019 | US | NA | TAUS:  Se 0.79-0.81, Sp 0.86-0.95 | overall US was accurate for the detection of postoperative SBCD, with a clear clustering of studies in the top left of the summary ROC | Low |

NA=not available, Se=sensitivity, Sp=specificity.

SBCD=small bowel Crohn’s disease

**Table S4:** General characteristic in diagnostic performance and clinical use of each tests

| **Non-invasive test** | **Summary of diagnostic performance** | **Advantages and disadvantages in clinical use** |
| --- | --- | --- |
| FC | The sensitivity and specificity of FC were balanced in general, and the sensitivity was slightly better than the specificity. The results obtained with different cut-offs were different. For diagnosis, FC performed better in distinguishing IBD from non-IBD (Se, 0.99; Sp, 0.65) and IBD from IBS (cut-off 50 μg/g: Se,0.97; Sp, 0.76; cut-off 100 μg/g: Se, 0.92; Sp, 0.86). In the diagnosis of IBD vs non-IBD, the performance of FC in CD patients (Se, 0.95; Sp, 0.84) was generally better than that in UC (Se, 0.78; Sp, 0.78) patients, however, the difference was not significant in activity assessment and prognosis. | Stools collection was moderately acceptable. Stools collection acceptability was impacted by the embarrassment to collect the stools, the dirtiness feeling, and the problem to transport the sample. The FDA defines FC as an immunologic test system intended for in vitro diagnostic use as an aid in the diagnosis of IBD. FC had a significant variability across platforms and collection techniques (point of care vs send out, commercial extraction vs manual weighting), and it was influenced by factors such as timing of collection within the day, physical attributes and characteristics of stool provided, and time between bowel movements (ie, stool frequency) |
| CRP | They all had good specificity for diagnosis, but the sensitivity of them was not good. As for assessing activity, only CRP were assessed. The prognostic effects of these indicators have not been studied. The specificity of CRP is more prominent in activity assessment (0.92) than in diagnosis (0.88). We only collected the diagnostic performance of ESR, PLT, Hb, Alb in the diagnosis of IBD vs non-IBD, among which Alb (0.94) had the best specificity. | Venipuncture was overall the most acceptable examination for patients |
| ESR |  |  |
| PLT |  |  |
| Hb |  |  |
| Alb |  |  |
| ASCA | It had good specificity for diagnosis, but the sensitivity of them was not good. The specificity of ASCA was good in both diagnosis of IBD vs non-IBD (0.925) and CD vs UC (0.892). Its effects in activity assessment and predicting relapse have not been studied. | Venipuncture was overall the most acceptable examination for patients |
| ANCA | ANCA also did well in differentiating IBD from non-IBD with high specificity (Se, 0.971). Its effects in activity assessment and predicting relapse have not been studied. | Venipuncture was overall the most acceptable examination for patients |
| FL | FL had great specificity in the diagnosis, whether in patients with IBD (0.95), UC (1.00) or CD (1.00). However, in the activity assessment (0.79), it did not perform well. | The tests for fecal lactoferrin rarely are used in routine clinical practice, probably because it is less stable at room temperature |
| antiGP2 | The specificity of Anti-gp2 is good both in the diagnosis of IBD vs non-IBD (0.96) and CD vs UC (0.97). Its effects in activity assessment and predicting relapse have not been studied. | Venipuncture was overall the most acceptable examination for patients |
| IGRA | The specificity of IGRA (0.867) was slightly better than the sensitivity (0.828) in distinguish CD from ITB, and the overall diagnostic performance of IGRA was better than that of ASCA (Se, 0.33; Sp, 0.83) in this clinical condition. Its effects in activity assessment and predicting relapse have not been studied. | The gold standard for differentiating ITB from CD is histology, smear test and culture, but the positive rate of them is very low. The sensitivity and specificity of smear test is not high. Besides, TB (tuberculosis) culture is very time-consuming. IGRAs is currently considered to be superior to tuberculin skin test, but due to the limitations of the test method, neither tuberculin skin test nor IGRAs can distinguish between TB latent infection and active TB. Therefore, CD with ITB or CD with latent infection of TB cannot be ruled out |
| FIT | The specificity of FIT (0.8) was slightly better than sensitivity (0.72) in activity assessment, but the overall performance was average. | Stools collection was moderately acceptable. Stools collection acceptability was impacted by the embarrassment to collect the stools, the dirtiness feeling, and the problem to transport the sample |
| MRE | The specificity of MRE in diagnosis and activity assessment was great in IBD patient and CD patient, among which DWI-MRE (Se, 0.929; Sp, 0.91) has the best overall performance. However, in predicting relapse, the sensitivity of MRE (0.973) was better. | In clinical use, MRE was a better-accepted tool for patients than stool collection. It doesn't have ionizing radiation, however it’s expensive and of limited availability |
| US | In the diagnosis of IBD vs non-IBD, US (0.95) had a high specificity. In terms of assessing activity, sensitivity and specificity were balanced, however, the sensitivity was great in predicting relapse, especially using SICUS (0.99). | Ultrasonography is the most acceptable examination for CD patients. Unfortunately, the operator-dependent characteristics and the difficulties to explore the overall bowel length are known limiting factors of ultrasound. Novel modalities of ultrasonography could counterbalance these weaknesses in the near future. Besides, comparing with MRI and CT, intestinal ultrasound has several advantages, including low financial cost, portability, availability, reproducibility, real-time assessment and the absence of radiation exposure and potentially nephrotoxic contrast agents |
| Scintigraphy | The diagnostic performance of scintigraphy was only available in the activity assessment, and LS (Se, 0.91; Sp, 0.85) performed better than MAAS (Se, 0.45; Sp, 0.94). | LS requires no intestinal preparation, has no side effects, has practical possibility even in the acute phase of IBD, and patients have less radiation exposure. But the process of labeling white blood cells in vitro is long and complex, not to mention the fact that cell labeling under sterile conditions requires trained staff and a well-equipped laboratory |
| CT | As for CT, the sensitivity and specificity were balanced in distinguish IBD from non-IBD and activity assessment. | CT can objectively show intestinal wall and extra-intestinal diseases, but its important disadvantage is radiation, which can make patients reluctant to be tested |
| miRNA | The sensitivity (0.80, 0.79–0.82) and specificity (0.84, 0.82–0.86) of miRNA in diagnosis of IBD were balanced and great. | Samples from peripheral blood, fresh tissues, or even FFPE tissues can be quickly and conveniently collected, and miRNA expression is consistent in these samples. Additionally, as a mature technique for miRNA testing, qPCR is simple and reliable. |
